# Luteolin: A promising natural agent in management of pain in chronic conditions

**DOI:** 10.3389/fpain.2023.1114428

**Published:** 2023-03-01

**Authors:** Foteini Ntalouka, Athina Tsirivakou

**Affiliations:** Department of Research and Development, Whisper P.C., Athens, Greece

**Keywords:** luteolin, inflammatory pain, neuropathic pain, chronic pain, pain managemant

## Abstract

Pain due to chronic conditions is a frequent and insufficiently addressed problem. Current drug options for pain management (either in cases of chronic inflammatory conditions or neuropathy) do not adequately treat pain. Moreover, they are associated with important adverse events in long term use. Luteolin is a flavonoid widely present in the plant kingdom and its sources have been assembled in a comprehensive list of this paper. Luteolin has shown in several research studies a range of pharmacological properties; anti-inflammatory, antioxidant, neuroprotective, and analgesic. In this article, we summarize the effects and potential benefits from introducing luteolin as an adjuvant agent in established protocols for pain management. We review the most indicative *in vivo* and *in vitro* evidence of how luteolin can target the molecular pathways involved in pathogenesis of chronic inflammatory and neuropathic pain. The data reviewed strongly support luteolin's promising benefits in pain management and raise the need for further clinical trials that can establish its role in clinical practice.

## 1. Introduction

Pain is defined by The International Association for the Study of Pain (IASP) as: “An unpleasant sensory and emotional experience associated with, or resembling that associated with, actual or potential tissue damage” (1).

Acute pain comes from the activation of the nociceptors to noxious stimuli. It works as a normal, warning system that protects the body from potential damage that can be caused by physical, thermal, or chemical threats. It can also result from a damaged (inflamed) tissue. In the latter case, pain is expected to be relieved when resolution of inflammation is achieved and the healing process is completed (2, 3).

**Figure 1:**
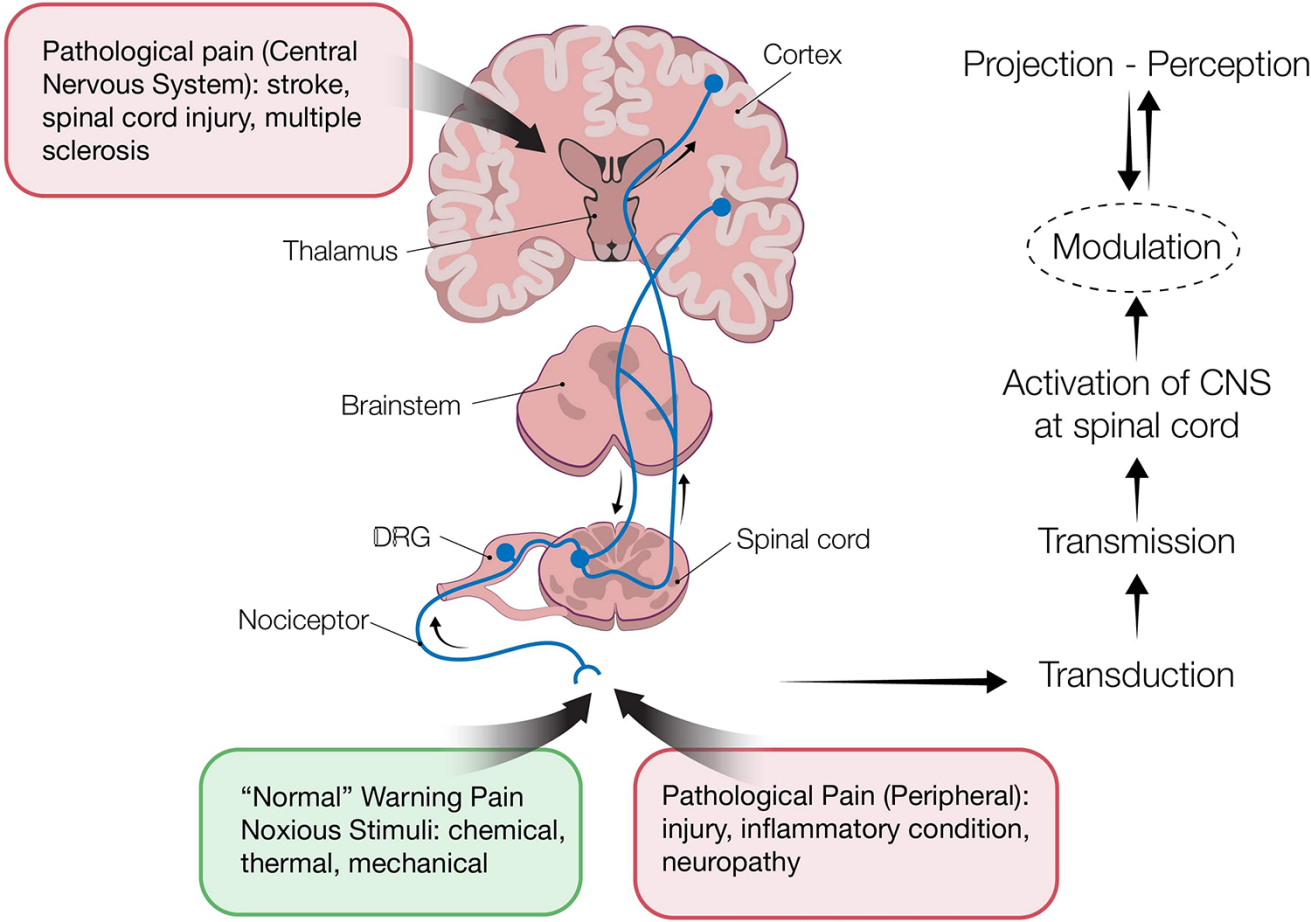
Pain pathways: transduction-transmission-perception-modulation. Transduction refers to activation of peripheral nociceptors (primary afferent neurons) from various stimuli. Cell bodies of nociceptors neurons are located in the dorsal root ganglia (DRG) (innervating the skin, deep tissues, visceral organs) and trigeminal ganglia (innervating the face). Nociceptors express transducers on their distal terminus, which are high-threshold ion channels, such as transient receptor potential ion channels [TRPs, ATP- gated ion channels, and acid-sensing ion channels (ASIC)]. These are responsible for converting the stimuli to action potential (AP). Next is the transmission process during which primary afferent neurons transmit the AP to the spinal cord, *via* their axons that terminate in the spinal dorsal horn (DH). Perception refers to the projection of pain signals in the brain, during which complex neuronal networks in the brain receive and “translate” from the spinal cord information about duration, location, and intensity of pain. Modulation is the process by which the nervous system either enhances or inhibits pain signals. Alteration of pain impulses occurs due to three endogenous mechanisms. 1. Segmental inhibition during which the inhibitory nerve in the spinal cord can be blocked to transmit noxious stimuli from C-fibers as a result of stimulation of the non-noxious Aβ fibers nociceptors (the “gate theory”). The method of pain management with transcutaneous electrical nerve stimulation is based on this theory. 2. The opioid system consists of opioid receptors in the brain, spinal cord and peripheral nerves that are activated by the binding of endogenous opioids (enkephalins, endorphins, and dynorphins). 3. The descending inhibitory nerve system, projecting *via* the midbrain, inhibits nociceptive transmission at the spinal cord dorsal horn.

Four processes occur during pain perception: Transduction, transmission, perception and modulation (Figure 1). The ascending pathway represents the signal transmission from peripheral nerves to the brain, whereas modulation can follow an ascending and/or descending pathway. Through modulation, pain impulses can be either enhanced or inhibited. Inhibitory mechanisms have been the basis for various pain control management approaches, e.g., opioids drugs that mimic the endogenous opioid inhibitory system. Neurotransmitters play an important role in the pain transmission and modulation. They can be either produced from peripheral nerves to facilitate pain transmission (e.g., serotonin released at the site of injury or inflammation) or to inhibit pain impulses (e.g., serotonin, GABA and opioids acting in spinal dorsal horn) (4, 5).

**Figure 2:**
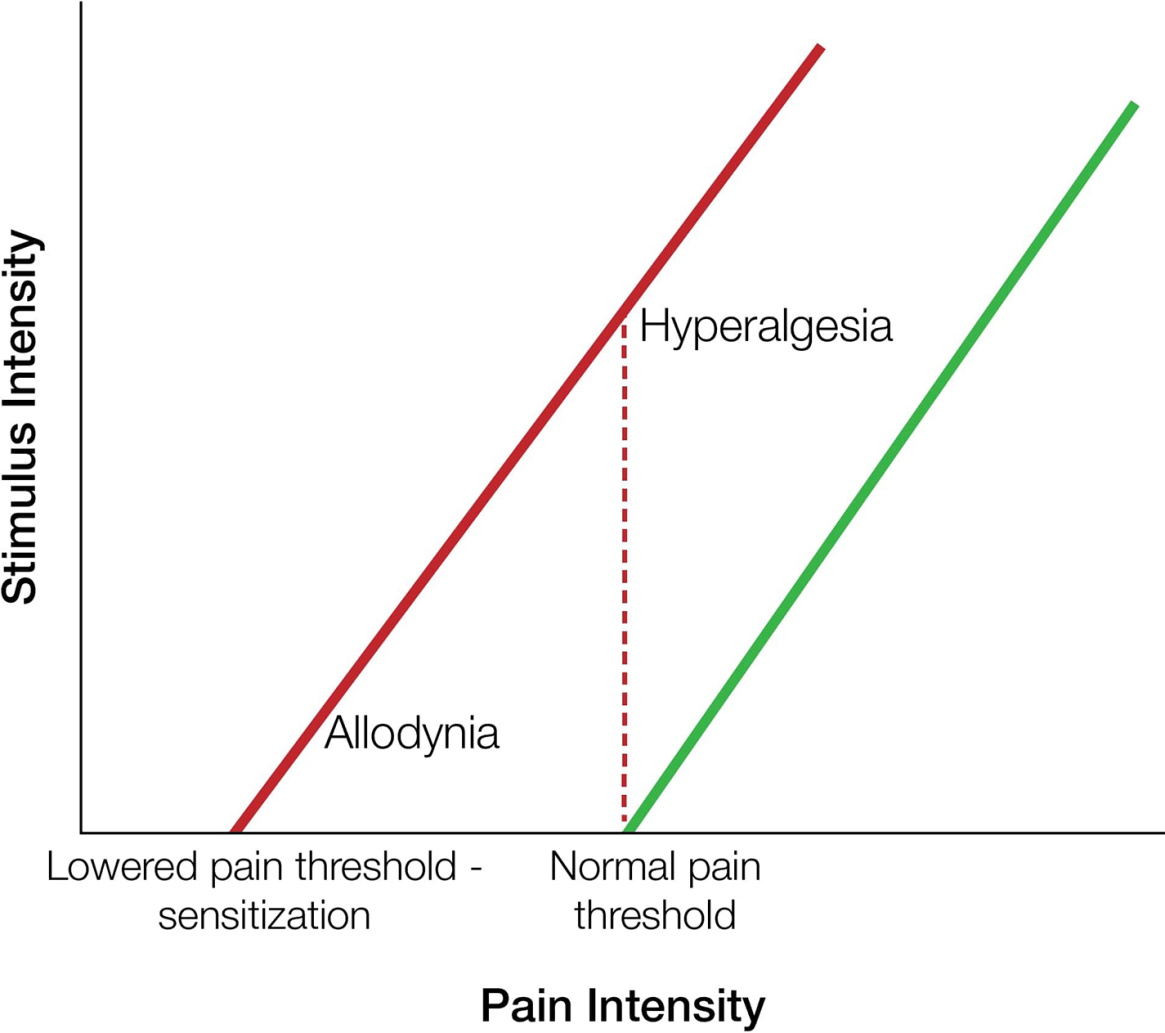
Allodynia and hyperalgesia vs. “normal” pain. Allodynia is the pain perception triggered by stimuli which under normal conditions are not painful. It is caused by a lowered pain threshold of nociceptors. Hyperalgesia is characterized by increased sensitivity to pain intensity triggered by normally painful stimuli.

In contrast to acute pain, pain in chronic disorders is a much more complicated state and may involve concurrently different mechanistic types of pain. It can be either nociceptive, neuropathic, or include both components. Nociceptive is defined as the pain caused by damage in non-neuronal tissues, whereas neuropathic pain is the pain resulting from damage in the neuronal system. Prolonged stimulation of the nervous system leads to sensitization, during which the threshold of nociceptors is reduced, and to the phenomenon of hyperalgesia (increased sense of pain from a stimulus that causes pain) or allodynia (pain provoked by a stimulus that normally is not painful) is induced (3) (Figure 2).

Experiencing pain due to chronic disorders can have a serious impact on the quality of life of the afflicted individuals. Sleep deprivation, stress, anxiety, and social withdrawal are common consequences which seem to also have a bidirectional relationship in pain perception (6).

Therefore, effective management of pain is crucial in chronic conditions and despite the advances in medical treatments, it remains a challenge in clinical practice. Maximizing effectiveness and minimizing the risks for side effects in long-term use, are the goals in treating pain. However, current drug options do not seem to completely achieve this goal, neither in targeting pain as a symptom of a chronic inflammatory disease, nor as a result of a neuropathic condition. Thus, the need for complementary approaches, which can be both effective and safe, has emerged (7, 8).

In the last decade natural compounds have gained much attention for their potential use in therapeutics. Flavonoids are a group of phytochemicals that is perhaps one of the most studied. They are a group of secondary metabolites of plants, found extensively in fruits, vegetables, and herbs. Due to their wide range of pharmacological activities and safety profile are considered to be promising agents against chronic inflammation and neuropathy (7, 8). Luteolin, a flavone that is abundant in a wide range of medicinal plants and herbs, has been reported to have a range of pharmacological properties. Among these, the anti-inflammatory, antioxidant (9, 10), neuroprotective (11), and analgesic (7) effects are the ones that can be of significant value in pain management.

In this article we will summarize the current knowledge about the activities of luteolin which bring out its potential in managing pain. We will focus on the two “classic” types of pain, chronic inflammatory-nociceptive and neuropathic, and how luteolin could target the mechanisms involved in their pathogenesis. The recently defined as nociplastic pain, referring to central nervous sensitization in the absence of obvious damage (as seen in fibromyalgia, irritable bowel syndrome, and medically unexplained conditions) (12) is not covered in this review, since the mechanisms implicated have not yet been adequately elucidated to support a beneficial role for luteolin.

## 2. Chemical structure, plant and dietary sources of luteolin

**Figure 3:**
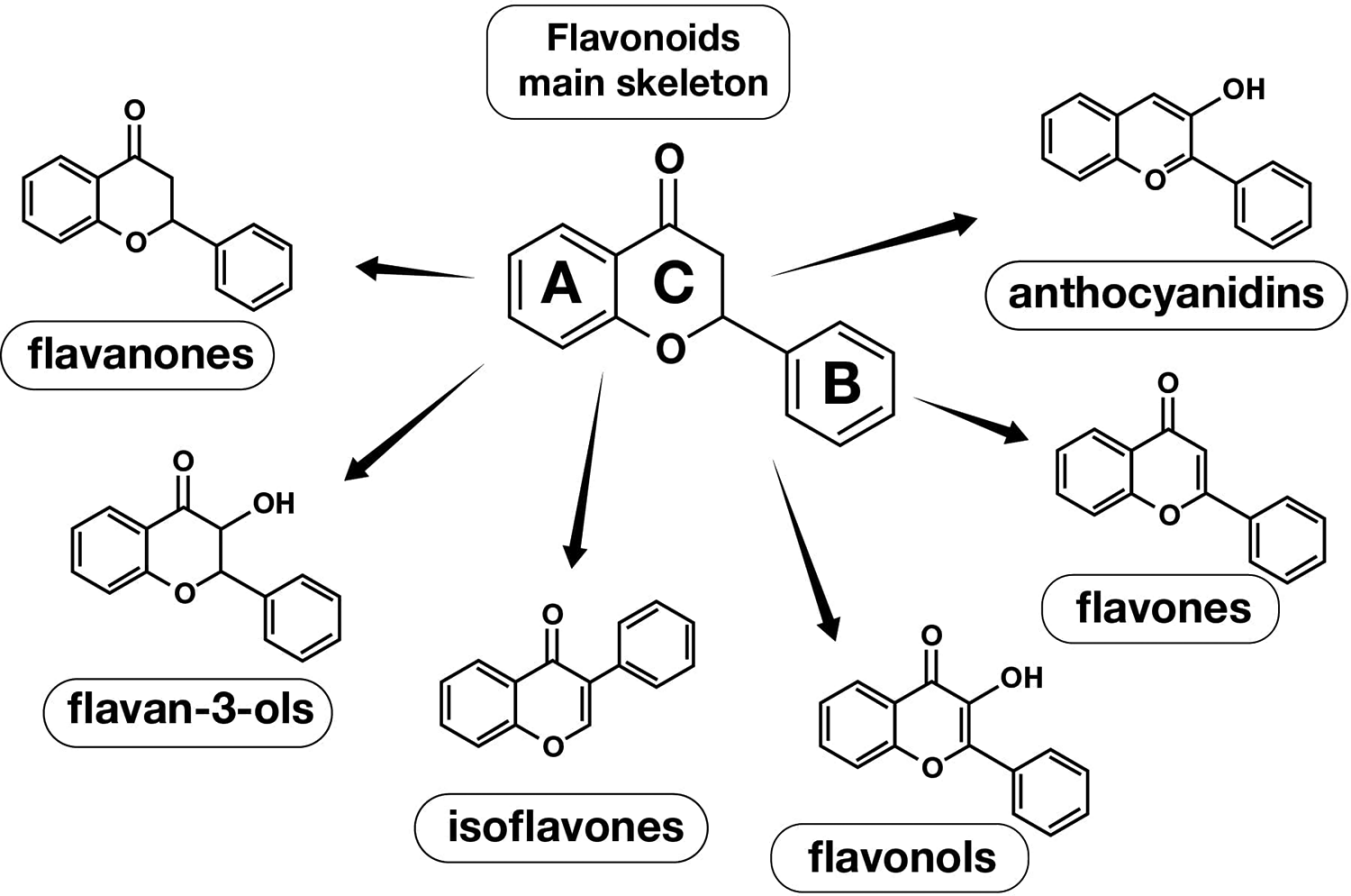
Flavonoids main skeleton and chemical structures of flavonoids different groups.

Luteolin belongs to a huge group of substances called flavonoids which are secondary metabolites characterized by a diphenylpropane structure (C6–C3–C6) and which are classified to many groups (Figure 3). They are divided into several subgroups based on structural differences in their ring C involving its oxidation state. The A and B-rings of flavonoids are usually functionalized by OH, OMe, isoprenyl, and glycosyl groups (13).

**Figure 4:**
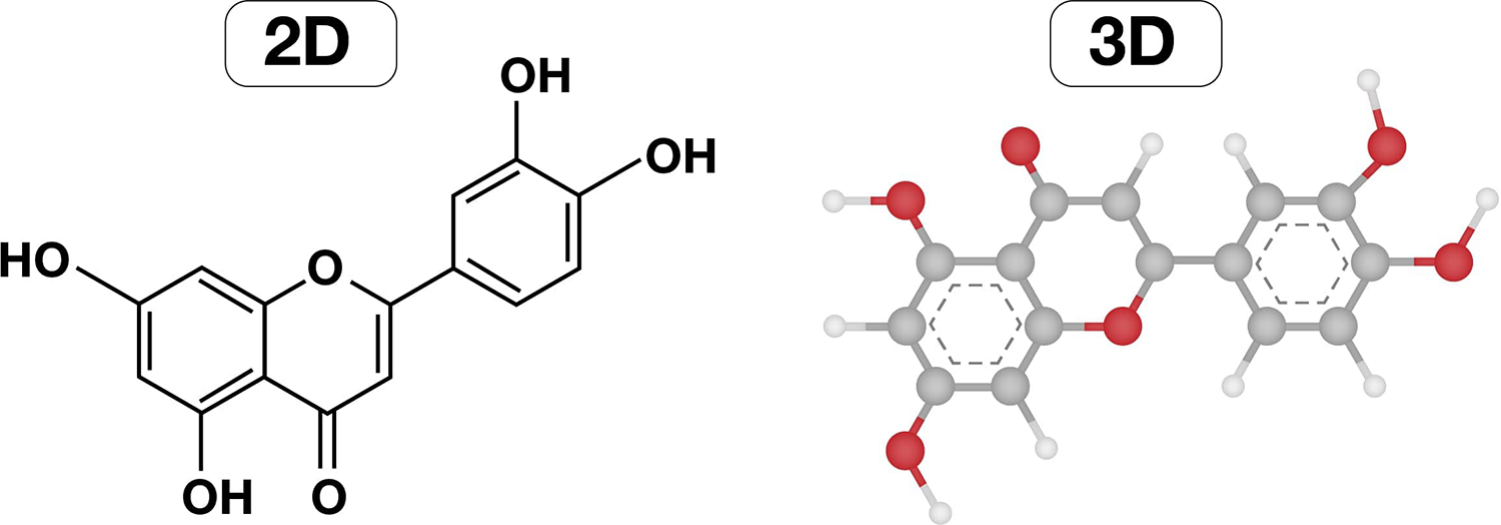
Chemical structure of luteolin in 2D and 3D.

Luteolin is a tetrahydroxyflavone in which the four hydroxyl groups are located in positions 3, 4, 5 and 7 as seen in Figures 4A,B. It is a flavone (3′, 4′, 5, 7-tetra hydroxyl flavone) with a yellow crystalline appearance. Due to its color, the plant *Reseda luteola* that contains luteolin, has been used as a source of dye from the first millennium B.C. Michel Eugène Chevreul, a France chemist, was the first who isolated luteolin in 1829, but the correct structure was proposed in 1896 by the English chemist Arthur George Perkin (14).

Luteolin is a substance found in several plant species, including those used in traditional medicine for the treatment of many diseases. It is widely distributed in the plant kingdom and has been studied extensively for its pharmacological properties, such as anti-inflammatory, antioxidant, and neuroprotective. Glycosides of luteolin have been identified in fossils of Ulmaceae species, 36 to 25 million years old. Over 350 plant species have been found to contain luteolin and/or its various glycosidic forms (Supplementary Table S1) (15).

Depending on the country, the daily intake of flavonoids in the human diet differs. The highest intake of flavonoids including polymers is in Ireland with a daily intake of 851 mg/day, while for the same group, (flavonoids including polymers) the lowest daily intake is 225 mg in the Czech Republic. Correspondingly, the lowest intake is in the Flavones with the highest being 10 mg/day in Italy, while the lowest is 2 mg/day in Sweden, Netherlands and the United Kingdom (Table 1).

**Table 1:** Daily intake (mg) of flavonoids in adults in the European union (16).

Countries/Flavonoids	Flavonols	Flavanones	Flavones	Flavonoids (monomeric compounds only)	Flavonoids (including polymers)
**Denmark**	19	13	3	128	379
**Finland**	17	30	3	134	354
**Sweden**	18	19	2	110	310
**Northern Region**	18 ± 1	21 ± 5	3	124 ± 7	348 ± 20
**Belgium**	19	12	3	97	281
**Czech Republic**	16	10	4	75	225
**Germany**	27	19	3	181	573
**Hungary**	23	11	4	122	408
**Ireland**	38	8	3	249	851
**Latvia**	25	4	9	135	411
**Netherlands**	31	18	2	201	643
**United Kingdom**	28	9	2	195	655
**Central Region**	26 ± 2	11 ± 2	4 ± 1	157 ± 21	506 ± 75
**France**	18	10	7	115	352
**Italy**	20	20	10	96	291
**Spain**	15	17	3	75	260
**Southern Region**	18 ± 1	15 ± 3	7 ± 2	95 ± 11	301 ± 27
**Europe**	23 ± 2	14 ± 2	4 ± 1	136 ± 14	428 ± 49

With regards to luteolin, although there are many plant sources (Supplementary Table S1), the daily intake in the European Union in adults ranges from 0 to 2 mg/day (Table 2) (16).

**Table 2:** Daily intake (mg) of individual compounds of flavonoids in adults in the European union (16).

Country/Flavonoids	Kampherol	Myricetin	Quercetin	Isorhamnetin	Hesperetin	Naringenin	Eriodictyol	Apigenin	Luteolin
**Denmark**	4	2	12	1	8	3	1	2	1
**Finland**	3	2	10	2	19	7	3	2	1
**Sweden**	3	1	12	1	12	5	2	1	1
**Northern Region**	4	2	11 ± 1	1	13 ± 3	5 ± 1	2 ± 1	2	1
**Belgium**	3	1	12	2	7	3	1	2	1
**Czech Republic**	4	1	9	3	6	3	1	3	1
**Germany**	6	2	17	2	13	4	1	2	1
**Hungary**	5	2	14	3	7	3	1	3	1
**Ireland**	11	4	21	3	5	2	1	3	1
**Latvia**	4	2	17	2	3	1	0	8	1
**Netherlands**	8	3	18	2	12	4	2	1	1
**United Kingdom**	8	3	16	2	6	2	1	1	1
**Central Region**	6 ± 1	2	15 ± 1	2	7 ± 1	3	1	3 ± 1	1
**France**	3	2	11	2	6	3	1	6	1
**Italy**	4	1	13	3	12	6	2	9	2
**Spain**	2	1	10	2	10	4	2	1	1
**Southern Region**	3	1	11 ± 1	2	9 ± 2	4 ± 1	2	5 ± 2	1
**Europe**	5 ± 1	2	14 ± 1	2	9 ± 1	4	1	3 ± 1	1

## 3. Luteolin in management of pain in chronic inflammatory conditions

### 3.1. Chronic inflammation

Pain, along with redness, warmth, swelling are the characteristic symptoms of inflammation. Inflammation is an evolutionary conserved and protective reaction of the body against factors that are threatening its normal functioning and homeostasis. Acute inflammation is a high-grade type of inflammation, triggered either by the presence of pathogenic microorganisms or cellular damages caused by noxious stimuli. Pathogen-associated molecular patterns (PAMPs) are activated in case of infections from pathogens, whereas damage-associated molecular patterns (DAMPs) are activated in case of cellular damage. These molecules are recognized from immune cells such as macrophages and dendritic cells (DC), which in turn are activated to produce inflammatory enzymes, such as cyclooxygenase 2 (COX-2) producing prostaglandin E2 (PGE2), and cytokines, such as tumor necrosis factor-alpha (TNF-α), interleukin 6 (IL-6), and interleukin 1-beta (IL-1β) (17). Recruited neutrophils are further promoting the inflammatory state. All the inflammatory mediators are playing a key role in acute inflammatory pain, through activating the nociceptors (17–19).

After a reasonable period of time and under normal conditions, when threat is eliminated and healing of the tissue has been completed, the resolution of inflammation will be achieved and relative symptoms including pain, will stop. Various factors, including environmental and genetic parameters, can contribute to preserved chronic inflammation, usually activated by DAMPs (sterile type). Persistent, non-resolving inflammation can lead gradually to detrimental effects in tissues and organs' normal structure and functioning. Pain can be one of the symptoms where the underlying pathology is a low-grade, progressive inflammatory state (3, 17, 18). Chronic conditions like osteoarthritis, rheumatoid arthritis, inflammatory bowel disease, are characterized by pathological and persistent pain. This type of pain results from sensitization of nociceptors to the inflammatory mediators' stimuli in the foci of inflammation, causing a shift from their high-threshold to low-threshold activation.

In the absence of cure, inhibition of inflammation is the gold standard approach in management of chronic inflammatory conditions, in order to achieve prolonged remission state, and consequently relief of pain. Non-steroidal anti-inflammatory drugs (NSAIDs), analgesics, corticosteroids, opioids, immunobiological molecules are being used in chronic inflammatory pain. Clinicians' choice is based on pathogenesis of the inflammatory condition itself, as well as on outweighing the effectiveness of the protocols designed and the risks for side effects. However, there is still a need for more effective and safe agents that can complement the current therapies (8).

### 3.2. Anti-Inflammatory properties of luteolin in chronic inflammatory pain

Luteolin, shows pleiotropic actions in various pathways involved in chronic inflammation, and due to its safe profile can be considered as a promising adjuvant therapeutic choice in inhibition of inflammation and related pain.

Luteolin has shown important effects in regulation of inflammation, in both *in vitro* and *in vivo* studies. Its actions are mostly exerted by inhibiting several biochemical pathways and inflammatory mediators, which are involved in several chronic diseases where prolonged inflammation is the common denominator of pathogenesis.

The anti-inflammatory activities of luteolin were comprehensively reviewed in literature in two review papers (9, 10). The reader can refer to these reviews for very detailed information on preclinical evidence about the anti-inflammatory effects of luteolin in various *in vitro* cell lines and *in vivo* animal models. Authors described extensively the regulatory effects of luteolin on inflammatory mediators such as cytokines IL-6, IL-1β, TNF-α, enzyme COX-2 and prostaglandins PGEs. Moreover, luteolin can inhibit the increased expression of inducible nitric oxide synthase (iNOS) and metalloproteinases (MMPs) in chronic inflammatory conditions (9, 10).

Nitric oxide (NO) has various roles under normal conditions. It promotes vasodilation, works as a neurotransmitter, and regulator of immune response. However, in chronic inflammation, an inducible form of NO is synthesized by synthase iNOS, leading to nitric oxide overexpression, even 1,000 times more than its physiological production. In this case, nitric oxide acts as an inflammatory mediator (20, 21).

MMPs are a family of metalloproteinases, and their primary action is to degrade extracellular matrix proteins. Although they have been attributed with many physiological roles in various essential functions, such as cell proliferation, tissue repair, wound healing, and others, they are also involved in inflammatory processes. In particular, increased expression of various types of MMPs have been implicated in tissue remodeling and destruction in chronic inflammatory conditions (22, 23).

**Figure 5:**
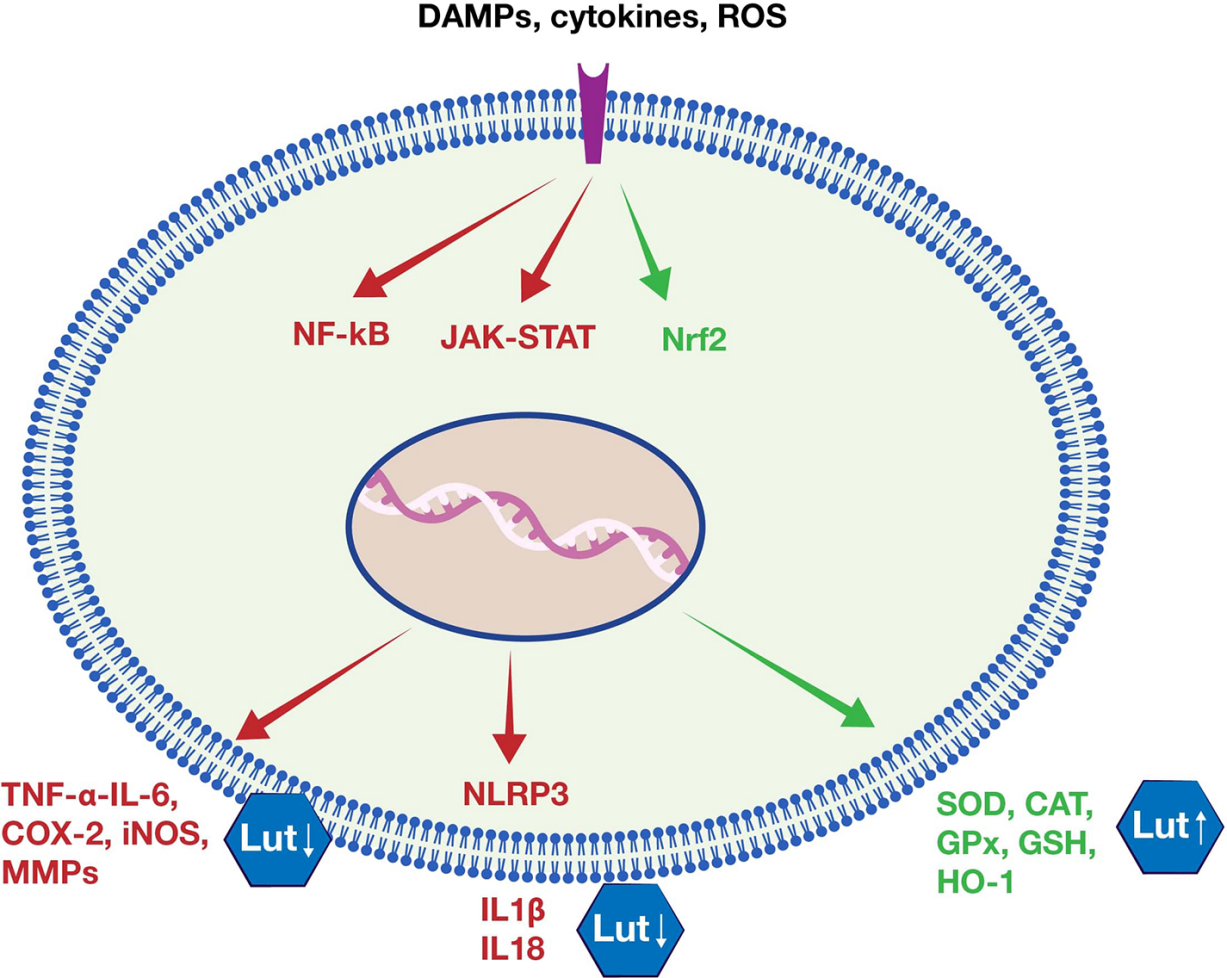
Luteolin's anti-inflammatory and antioxidant effects. Luteolin inhibits major inflammatory signaling pathways (e.g., NF-κΒ, JAK-STAT, NLRP3), leading consequently to reduced expression of pro-inflammatory mediators (e.g., TNF-α, IL-6, COX-2, iNOS, MMPs, IL1β, IL18). Moreover, luteolin seems able to activate the major antioxidant factor Nrf2, and increase the expression of antioxidant enzymes (e.g., SOD, CAT, GPx, GSH, HO-1).

The inflammatory response requires the orchestrated activation of different but interacting signaling pathways. Among of those, nuclear factor kappa B (NF-κΒ), Janus kinase - signal transducer and activator of transcription (JAK-STAT), and inflammasome NOD-like receptor (NLR family) pyrin domain containing 3 (NLRP3) play important roles in expression of pro-inflammatory genes. NF-κB is considered a master transcription factor in both acute and chronic inflammation, involved in expression of pro-inflammatory cytokines, chemokines, adhesion molecules, iNOS, and metalloproteinases (MMPs) (24). JAK-STAT is another signaling pathway whose activation is implicated in auto-immune and inflammatory disorders. It is used as a signal transduction pathway from cytokines to promote the inflammatory response and it relates to NF-κΒ signaling pathway activation (25). NLRP3 is the most studied inflammasome, a multiprotein complex whose oligomerization activated by PAMPs or DAMPs, leads to maturation of the potent immune modulators IL-1β and IL-8 (26). Research studies showed that luteolin can beneficially regulate these pathways during the inflammatory process (10, 27–29), as summarized in Figure 5.

Based on its multi-targeted anti-inflammatory actions, luteolin appears to be a very promising natural agent for inhibiting abnormal inflammatory responses in chronic inflammatory conditions. In Table 3, we provide a summary of recent *in vitro* and *in vivo* studies, reflecting this flavonoid's potential effects on conditions characterized by significant pain during flares of the inflammatory diseases: Rheumatoid Arthritis, Osteoarthritis, and Inflammatory Bowel Disease.

**Table 3:** Effects of luteolin on chronic inflammation as shown from *in vitro* and *in vivo* studies.

*in vitro*/*in vivo*	Cell line/Animal model	Concentration/Dose and route of adminstration of luteolin	Effect	Refs
**Rheumatoid Arthritis**				
*in vitro*	Human synovial sarcoma cell line, SW982	1 or 10 μM	Inhibited IL-1b-induced MMPs (MMP-1 and -3), cytokines TNF-a and IL-6	(27)
	Rat FLS cells (RSC-364)	luteolin 25 μmol/l in combination with Chlorogenic acid	Inhibited the proliferation of fibroblast-like synoviocytes	(30)
	LPS-induced RAW 264.7 macrophages	0.25–0.5 μM	Inhibited iNOS, IL-6 and TNF-α	(31)
*in vivo*	Collagen Induced Arthritis (CIA) mice	co-ultramicronized PEA/Palmitoylethanolamide + luteolin 1 mg/kg, intraperitoneally	Reduced neutrophil and mast cell infiltration into the joints, improvements in locomotor activity and pain sensitivity	(32)
	(FCA)-induced arthritis (AA) in male rats	10 and 20 mg/kg intragastrically	Reduced, TNF-α, IL-6, IL-1β and IL-17, alleviated synovial hyperplasia, protected from joints destruction	(33)
**Osteoarthritis**				
*in vitro*	rabbits cultured articular chondrocytes	100 μM	Inhibited IL-1β MMP3 induced expression, and other degradative enzymes	(34)
*in vivo*	knee joint of rats on *in vivo* IL-1b-stimulated production of MMP-3 from articular cartilage tissues.	100 μM injection into the right knee joint	Inhibited IL-1b-induced production level of MMP3	
*in vitro*	interleukin (IL)-1β-stimulated rat chon- drocyte	25, 50, 100 μΜ	Reduced iNOS, NO, PGE2, COX-2, TNF-α, MMP1, MMP2, MMP3, MMP-8, MMP-9, MMP13, reversed collagen II degradation	(35)
*in vivo*	MIA-induced mice model of OA	10 mg/kg daily by gavage	Alleviated articular cartilage destruction	
**Inflammatory Bowel Disease**				
*in vitro*	Human mast cells - HMC-1	50 μM	Inhibited TNF-α, IL-8, IL-6, GM-CSF, and COX-2	(36)
	Human colon epithelial cells - HT-29	50 and 100 μM	Inhibited IL-8, COX-2, iNOS	(28)
*in vivo*	DSS-induced colitis mouse model	8 mg/kg oral administration	Inhibited IL-17 pathway	(37)
	*DSS-induced colitis mouse mode*	50 mg/kg by gastric gavage	Reduced IL-1β IL-6	(38)

Antioxidant properties of luteolin could also contribute to inhibition of chronic inflammation. Oxidative stress and inflammation are closely related. In fact, evidence shows that there is a bidirectional relationship between excessive free radicals' production and inflammation. Chronic inflammatory conditions are characterized by oxidative stress, and vice versa. Excessive production of free radicals is implicated in the pathogenetic triggers of chronic inflammation: It leads gradually to accumulation of damages to fundamental components of cells, such as DNA, lipids and proteins, and induces activation of inflammatory pathways, such as the NF-κΒ (39, 40).

Flavonoids are potent antioxidant agents (41). Luteolin has shown important antioxidant actions, acting directly, *via* its ability to scavenge free radicals, but also by inducing endogenous antioxidant cell defenses. In various *in vitro* and *in vivo* experiments, it was found that luteolin promotes increased production of antioxidant enzymes (9). One of the main pathways involved in this pathway is suggested to be the activation of the nuclear factor erythroid 2-related factor 2 **(**Nrf2) pathway (Figure 5) (42).

More detailed information on Luteolin's antioxidant capacity will be discussed in the next section about Neuropathic pain, where oxidative stress seems to play a primary role in pathogenesis.

## 4. Luteolin in management of neuropathic pain

### 4.1. Neuropathic pain

Neuropathic pain is the type of pain that results from damage or disease of the somatosensory nervous system. Its origin can be either peripheral (e.g., peripheral nerve injury pain, chemotherapy induced, cancer pain, diabetes peripheral neuropathy, alcoholic neuropathy) or central (e.g., spinal cord or brain injury, stroke, neurodegenerative disease). Though the pathogenetic mechanisms have not been fully elucidated, there is growing evidence that nitrosative and oxidative stress, along with neuroinflammation, have crucial roles in neuropathic pain.

Oxidative stress and nitrosative stress represent the loss of redox balance in the cells, due to high levels of reactive oxygen species (ROS) and reactive nitrogen species (RNS) respectively. ROS, produced by nicotinamide adenine dinucleotide phosphate (NAPDH) oxidase (NOX) enzymes, and RNS, produced by iNOS enzymes, are generated constantly during normal metabolic cellular processes. When in low concentrations they have various protective roles, e.g., contributing to a normal immune response, but they are potentially detrimental when highly increased. ROS and RNS can easily react with other molecules, causing important oxidative damages to components which are fundamental for cells' healthy functioning and survival (lipid peroxidation, protein and DNA oxidation) (43–45).

Cells normally have endogenous defenses to maintain concentrations of these reactive species under control (redox balance), including a series of antioxidant enzymes, such as superoxide dismutases (SODs), catalase (CAT), glutathione (GSH), heme oxygenase 1 (HO-1). The transcription factor Nrf2 is considered the governor for the expression of more than 200 antioxidants enzymes. However, this antioxidative ability can be compromised as a result of disease or injury, due to excessive production of ROS and NOS (45, 46).

Nerve cells are more vulnerable to nitro-oxidative damage, because they have weaker antioxidant defenses mechanisms and higher lipid content, compared to other types of cells. Therefore, nitro-oxidative stress in neurons can be considered as a primary cause of neuropathic pain. It can lead to mitochondrial dysfunction - further increasing ROS levels - and induce pro-inflammatory cytokines production. Moreover, ROS have been involved in the enhancement of excitatory signaling of nociceptive nervous cells. They can induce excitatory N-methyl-D-aspartate **(**NMDA) receptors, inhibiting glutamatergic regulation, and contribute to reduction of GABA-ergic inhibitory signaling. In addition, ROS seem to contribute to activation of the ion channels TRP, which are highly expressed in nociceptors neurons and have an important role in transduction of pain (44, 47).

Apart from damage caused directly by ROS and NOS, neuroinflammation seems to be a key process in induction and maintenance of neuropathic pain. Neuroinflammation is the type of inflammation triggered by damage of neuronal tissue in the peripheral or central nervous system. When the peripheral nerve is damaged, resident immune cells (e.g., mast cells and macrophages), along with immune cells recruited from blood circulation (e.g., neutrophils and T-cells) release inflammatory mediators, such as cytokines IL-1b and TNF-a, prostaglandins, and ROS. Moreover, microglia and astrocytes in the spinal dorsal horn are activated, which in turn contribute to the release of inflammatory mediators in the central nervous system and enhancement of excitability. This cascade of inflammatory events leads to peripheral and central sensitization (44, 48).

Both inflammation and nitro-oxidative stress shall not be considered as independent processes in neuropathic pain, as they rather interact in a bidirectional relationship.

Excessive ROS and RNS induce neuronal inflammatory response and vice versa. For example, the transcriptional factor NF-κΒ is one of the most characteristic examples of an inflammatory biochemical pathway that can be activated by ROS/RNS leading to increased expression of inflammatory mediators, but also itself can result to increased nitro-oxidative species production (44, 48, 49).

Pharmacological options currently available for neuropathic pain target the pain as a symptom, rather than inhibit the causes of its pathogenesis. First line drugs include tricyclic antidepressants, serotonin-norepinephrine reuptake inhibitors, gabapentin and pregabalin. Second-line therapy includes tramadol, capsaicin patches, and lidocaine patches. Last line of choice are strong opioids. Due to their inadequate efficacy and important side effects in long-term use of these drugs, effective management of neuropathic pain seems to be quite difficult. An outcome of 30%–50% pain reduction is considered meaningful (50, 51), with a significant number of patients, even >50% in relative surveys, reporting insufficient management of their painful symptoms (52, 53).

Direct antioxidants (e.g., Vitamins C, E, coenzyme Q) have been tried but clinical outcomes failed to support their use (α-Lipoic acid seems however to have therapeutic efficacy in case of diabetic neuropathy). It is worth noting that apart from their unfavorable pharmacokinetic and pharmacodynamic profile, another possible reason for the failure of direct antioxidants is that they may interfere with the physiological (and protective) functions of ROS (44, 45).

Newer and safer approaches that could target selectively multiple pathways may be more effective, protecting from nitro-oxidative damage and at the same time inhibiting neuroinflammation.

### 4.2. Neuroprotective and analgesic properties of luteolin in neuropathic pain

**Figure 6:**
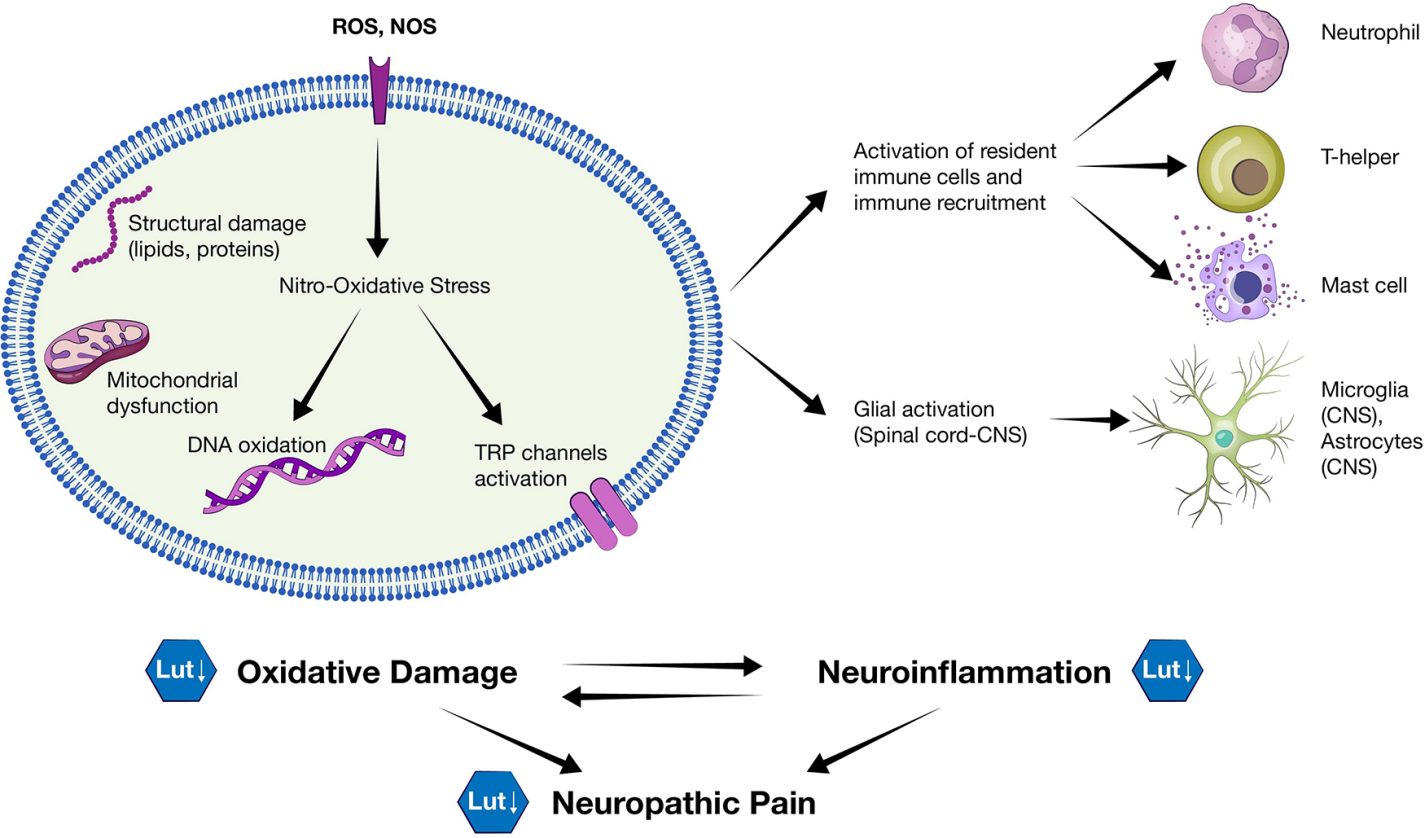
Oxidative damage and neuroinflammation in neuropathy. An illustration of the pathogenetic mechanisms implicated in neuropathic pain. ROS and NOS can induce nitro-oxidative damage in the vulnerable neuronal cells and activation of TRP channels which are involved in neuropathic pain transduction. Neuroinflammation - induced from neuronal cell damage - involves the activation of resident immune cells and glial activation. Oxidative damage and neuroinflammation, which are considered to be reciprocal processes, can be inhibited by luteolin.

Luteolin is a very promising agent against neuropathic pain, due to its effects on two molecular mechanisms involved in neuropathy pathogenesis: oxidative stress and inflammation. Its potent antioxidant abilities, its actions against neuroinflammation, along with its analgesic effects, justify luteolin as a possible complementary therapeutic compound in neuropathy (Figure 6).

Luteolin's antioxidant properties are well established. It works as a direct free radical scavenger, due to the presence of the hydroxyl groups in the B ring that act as an electron-donating system. In addition, it has been shown that luteolin can induce the enhancement of endogenous antioxidant capacity. It can lead to increased expression of antioxidant enzymes, such as SOD, CAT, glutathione peroxidase (GPx), GSH, HO-1, NADPH quinone dehydrogenase 1 (NQO1). This particular action is of very special interest in the case of neuropathic pain (9, 42, 54–56). As mentioned previously, direct antioxidants may not be as effective as expected, and current research has turned its focus on agents that induce endogenous antioxidant mechanisms, rather than just neutralize directly the free radicals. Luteolin seems to achieve this action by activating the major antioxidant transcription factor Nrf2. Moreover, luteolin has been extensively researched for its neuroprotective effects. It appears that it can cross the blood-brain barrier and exert important antioxidant and anti-inflammatory effects in the brain (57–59).

In various preclinical studies, it has been shown that luteolin has a pleiotropic function against mechanisms involved in neurodegenerative diseases, such as Alzheimer's, Parkinson's, and Multiple Sclerosis. It can prevent the activation of microglial and astrocytes, the upregulation of inflammatory mediators, such as cytokines and iNOS, and can also reduce oxidative stress. Again, the major signaling pathways on which Luteolin acts, inducing this anti-inflammatory and antioxidant profile, include the inhibition of NF-κ*Β* and induction of Nrf2 respectively, among others (11, 60, 61).

Even though the neuroprotective role of luteolin in the central nervous system is well established from *in vitro* and *in vivo* studies, its benefits on peripheral neuroinflammation still lack evidence. However, as one would notice from the present review, the pathways involved in inflammatory response and oxidative damage are universal, independently of the tissue damaged. Therefore, we can suspect that luteolin will most likely act beneficially also against damages of the peripheral nervous system. In fact, the limited but important *in vivo* studies about luteolin's effect on neuropathic pain are very encouraging (Table 4).

**Table 4:** *In vivo* studies of luteolin's analgesic effects in neuropathic models.

Animal model	Dose and route of administration of luteolin	Effect	Refs
male Sprague–Dawley rats	intrathecally administered luteolin 1.5 mg - intraperitoneally administered luteolin 50 mg/kg	Attenuated mechanical and cold hyperalgesia	(62)
diabetic rats - neuropathy induced by intraperitoneal injection of streptozotocin in rats	intraperitoneally 50 mg/kg, 100 mg/kg and 200 mg/kg	improved the impaired nerve functions, activated Nrf2, increased activities of antioxidant enzymes SOD, GST, GPx and CAT, and reduced ROS production, attenuated hyperalgesia and allodynia in a dose dependent manner	(63)
sciatic nerve ligated mice luteolin	intraperitoneally 5 mg/kg and 10 mg/kg body weight	reduced hyperalgesia in acute and chronic phase of formalin test	(64)
Lewis's lung cancer induced bone pain in mice	intraperitoneally 10 mg/kg and 50 mg/kg	ameliorated pain in the pain behavior test in a dose-dependent manner, inhibited activation of glial cells in spinal dorsal root and NLRP3 inflammasome	(65)

Luteolin could attenuate in a dose dependent manner, the mechanical and cold hyperalgesia in an animal model of neuropathic pain (62). The researchers also investigated the mechanism of its antinociceptive action. Antihyperalgesic effects of luteolin (intrathecally administered luteolin 1.5 mg or intraperitoneally administered luteolin 50 mg/kg) were inhibited by intrathecal pretreatment with the γ-aminobutyric acid A (GABAA) receptor antagonist bicuculline and μ-opioid receptor antagonist naloxone, but not by intrathecal pretreatment with either the benzodiazepine receptor antagonist flumazenil or glycine receptor antagonist strychnine.

In a diabetic neuropathy model, luteolin was able to improve the impaired nerve functions (63). Intraperitoneally daily treatment with luteolin (50 mg/kg, 100 mg/kg and 200 mg/kg) induced activation of Nrf2, increased activities of antioxidant enzymes SOD, GST, GPx and CAT, and reduced ROS production. Nerve function, as measured by increase of motor and sensory conduction velocities were also improved with luteolin treatment. Moreover, mechanical withdrawal threshold, cold, and heat withdrawal latencies were increased, indicating that luteolin can attenuate hyperalgesia and allodynia. All results were induced in a dose dependent manner, with doses of 100 mg/kg and 200 mg/kg of luteolin treatment leading to a significantly better outcome than dose of 50 mg/kg.

Administration of luteolin dose (5 mg/kg and 10 mg/kg body weight) significantly reduced neuropathic pain in another animal model. Interestingly, co-administration of luteolin and morphine (1 mg/kg), potentiated morphine analgesic effects (64).

Intraperitoneally treatment with luteolin ameliorated Lewis's lung cancer (LLC)-induced bone pain in mice in a dose-dependent manner (65) (50 mg/kg significantly improvement in pain behavior, than 1 mg/kg and 10 mg/kg). Bone cancer pain (BCP) is a severe complication in cancer patients with both inflammatory and neuropathic components. Intrathecal injection of luteolin 0.1 mg also reduced BCP in this study. Further analysis about the mechanism of action, showed that luteolin inhibited important components of neuroinflammation, such as activation of glial cells in spinal dorsal root and NLRP3 inflammasome.

## 5. Discussion

Pain management, especially in chronic conditions, remains a major challenge for clinicians. In case of a chronic inflammatory disorder, the goal of treatment is to inhibit the inflammatory process, prolong, and increase frequency of remission stages, and consequently reduce pain symptoms. In neuropathic pain, management is mainly symptomatic, targeting the pain as a symptom rather than the pathogenetic mechanisms involved. In either case, we are looking mostly at management rather than therapeutic approaches. Moreover, the drugs used are associated with important side effects in long term use, which raises the need for more efficient and safer options (8).

In this paper, we reviewed the potential beneficial effects of a flavone, luteolin, in pain management. Luteolin has potent anti-inflammatory actions, inhibiting important mediators of inflammation, that are also involved in pain, such as cytokines (e.g., TNF-a, IL-1, IL-6, IL-8) and enzymes (COX-2, iNOS). Luteolin also exhibits strong antioxidant activity, acting as a scavenger of free radicals, but also inducing expression of endogenous antioxidant enzymes (e.g., SOD, CAT, GPx, GSH) (9, 10). Since inflammatory process and nitro-oxidative damage are key mechanisms in both chronic inflammation and neuropathy, luteolin shows favorable effects in management of these conditions.

Moreover, the antinociceptive effects of flavonoids, including luteolin and its derivatives, have been observed in experiments even from earlier decades (7, 66–68). The mechanism of luteolin's effect on pain modulation process still remains unclear. There have been indications from *in vitro* and *in vivo* studies about its possible activity in GABA and opioid receptors. As mentioned previously in this review, analgesic effects of luteolin in a rat neuropathic model were inhibited by pretreatment with the γ-aminobutyric acid (GABAA) receptor antagonist bicuculline and μ-opioid receptor antagonist naloxone, but not by pretreatment with either the benzodiazepine receptor antagonist flumazenil or glycine receptor antagonist strychnine (62). The proposed mechanism that luteolin may act on GABAA receptor in a bicuculline-sensitive and flumazenil-insensitive manner has been further supported (69, 70). However, further studies are required to provide strong evidence on the involvement of luteolin effect on the GABAergic system and its activity profile at GABAA receptor subtypes. It will also be interesting to investigate any possible activity of luteolin on pain transmission, e.g., *via* modulation of ion channels activity, such as TRP, since other flavonoids have shown such effects (71).

However, from research studies to actual establishment luteolin as a therapeutic agent there are crucial parameters that need to be examined and fully clarified.

Bioavailability is one of these important factors. This measurement identifies the proportion of the substance that is available in the systemic circulation, taking into account the intestinal absorption and first-pass metabolism. Flavonoids, in general, are well known for their low oral bioavailability, because of their low water solubility and their rapid, and extensive first-pass metabolism. However, this does not necessarily reflect a low bioactivity. There is an evidence-based theory trying to explain the paradoxical phenomenon for polyphenols’ high bioactivity, in contrast to their low bioavailability. According to this theory, polyphenols, including flavonoids, could also exert their beneficial effects through their metabolites. In addition, prolonged bioavailability due to flavonoids' enterohepatic recycling, can contribute to their bioactivity (72, 73).

Luteolin, after its efficient absorption in intestinal epithelium, is extensively metabolized *via* glucuronidation, sulphation, or methylation. Research data (*in vivo* and in humans) have shown that after oral administration, luteolin presents in systemic circulation mainly in the form of luteolin conjugates, with more abundant being the luteolin glucuronides and luteolin sulfates (74, 75). Apart from the known bioactivity of free luteolin, its metabolites seem to be also biologically active, with evidence on their anti-inflammatory activity (76, 77). Another interesting process that was identified in a study, is the hydrolysis of luteolin monoglucuronide to luteolin at the site of inflammation. *β*-glucuronidase released from neutrophils as a result of the inflammation, was able to transform the luteolin glucuronide conjugates to free luteolin (78). Moreover, there are indications for the enterohepatic recirculation of luteolin, as studies have shown a second peak of their concentration and prolonged presence of luteolin and its metabolites in plasma (74, 77). From these data we can conclude that despite luteolin's low bioavailability (e.g., ranging from 4,1% at dose of 50 mg/kg (79), to 17,5% at dose 200 mg/kg (77) in rats), luteolin metabolites and recycling can contribute to luteolin's bioactivity.

In any case, a variety of methods have been tried successfully to enhance water solubility, bioavailability, and efficacy of luteolin. For example, complexes of luteolin with cyclodextrin (physicochemical data) (80) and phospholipids (physicochemical and *in vivo* evidence) (81, 82) have shown promising results. Moreover, nanoencapsulation of luteolin in liposomes (83, 84), micelles (85) and nanoparticles (86) as well as utilization of a microemulsion system (87) were able to improve *in vivo* luteolin's bioavailability and efficacy. Undoubtedly, there is a need for clinical studies proving these effects in humans.

To increase the effectiveness of luteolin against inflammatory and neuropathic pain, an interesting approach is to investigate a possible synergistic action in combination with other flavonoids. Quercetin is one of the most studied flavonoids, including *in vitro*, *in vivo*, and human studies. Like luteolin, quercetin shows potent antioxidant (88), anti-inflammatory (89), and neuroprotective (90) actions. Clinical trials have provided promising evidence on quercetin's beneficial role in alleviating chronic inflammation (91). A randomized, double-blind, placebo-controlled study investigated quercetin effects (500 mg/day) in 50 women with rheumatoid arthritis. Inflammation, stiffness and pain significantly reduced (92). Moreover, extensive preclinical research supports quercetin's protective actions against neuroinflammation and neuropathy. In addition, quercetin exerts analgesic effects (93). Examples of other flavonoids, well known for their anti-inflammatory, antioxidant, and analgesic properties are apigenin, rutin, naringenin, genistein (8, 94).

Even though there is already strong evidence on the anti-inflammatory, antioxidant, and neuroprotective effects of luteolin from preclinical studies, results from clinical trials are only scarce. However, beneficial outcomes of these studies pave the way for further investigation of luteolin's effects on patients suffering from chronic inflammatory conditions and/or neuropathies.

A nutritional supplement containing a mixture of flavonoids, luteolin (100 mg), quercetin (70 mg), and rutin (30 mg), has shown to improve clinical outcome in children with autism spectrum disorder (ASD). Treatment with 2 capsules/20 kg of body weight for at least 4 weeks led to significant improvement in gastrointestinal and allergy symptoms in about 75% of the children, eye contact and attention in 50%, social interaction in 25%, and resumption of speech in about 10% (95). These results were followed by another clinical study, during which treatment with 1 capsule/10 kg of body weight for 26 weeks, resulted in significant benefit in ASD children both in adaptive functioning and behavioral difficulties (assessed by the Vineland Adaptive Behavior Scales, and Aberrant Behavior Checklist respectively) (96). Researchers collected and analyzed a series of serum inflammatory markers from the children with ASD who participated in the latter study, before and after treatment with luteolin supplementation. Control samples were gathered from healthy children unrelated to the ASD subjects. The analysis showed that cytokines TNF-α and IL-6 were elevated in ASD patients compared to controls, and treatment with the formula containing luteolin led to a significant decrease of these inflammatory indices. The decrease was observed in the children with ASD whose behavior improved the most after supplementation (97). It is worth noticing that this is the first study that provided clinical evidence about luteolin's effects on inflammatory markers in relation to a beneficial clinical outcome.

Another interesting observation is the potential synergistic and beneficial effect of luteolin with Palmitoylethanolamide (PEA). Preclinical and clinical studies have reported an anti-inflammatory and analgesic action of PEA, a naturally occurring lipid mediator molecule (98). A two-arms study was performed to investigate the neuroprotective effects of a co-ultramicronized composite containing PEA and luteolin (co-ultraPEALut). In the first arm, rats subjected to middle cerebral artery occlusion and treated with co-ultraPEALut showed reduced edema, improved neurobehavioral functions, and reduced neuroinflammation after treatment. In the second arm, researchers examined the effects of a pharmaceutical co-ultraPEALut preparation treatment for 60 days on a cohort of 250 stroke patients undergoing neurorehabilitation. At study end, indices of neurological status, cognitive abilities, degree of spasticity, pain, and independence in daily living showed significant gains (99). Despite this being an observational rather than a controlled clinical trial, it adds clinical evidence on luteolin's beneficial effects.

In efforts to manage pain in chronic conditions we shall also consider that anxiety and depression are frequent comorbidities. In fact, there is evidence that a bidirectional relationship exists between them. Increased pain perception may result because of a chronic condition, and vice versa. A chronic painful condition increases the risk for psychopathological disorders (6, 100, 101). Flavonoids, including luteolin, have shown anxiolytic actions (102, 103). Characteristic example is the case of *Chamomilla matricaria*, a herb used traditionally for centuries in anxiety and depression. Its anxiolytic effects are attributed to its content in the flavonoid apigenin (104, 105).

Apart from its bioavailability and efficacy, safety is also of utmost importance when considering any compound for therapeutic purposes. Flavonoids are generally considered quite safe compounds even in doses largely exceeding daily dietary intake (106). Oral administration of luteolin has shown LD50 values even above 5,000 mg/kg (10, 106). In the clinical trials mentioned previously, safety of oral supplementation with luteolin was also recorded (95–97, 99). However, there is still a need for further toxicological studies to fully determine luteolin safety profile.

This review summarized the key properties of luteolin englighting its potential in management of pain in chronic conditions.

• Luteolin appears to be an excellent candidate for alleviating pain in chronic inflammatory conditions (e.g., rheumatoid arthritis, osteoarthritis, inflammatory bowel disease), inhibiting major inflammatory mediators involved in manifestation of pain as a symptom of the disease.
• Based on its strong anti-inflammatory and antioxidant properties shown in preclinical studies, luteolin can inhibit the major components of pain pathogenesis in neuropathy, namely oxidative stress and neuroinflammation, that lead to nerve damage and chronic pain.
• Moreover, as we described previously, there is evidence that it can also show analgesic effect *via* interaction with GABAA receptors.
• Luteolin appears (from preclinical and clinical data) to have a very good safety profile, making it even more appealing for clinical implementation.

However there are limitations before it can be further developed into an analgesic drug for pain management. Additional studies in chronic inflammatory and neuropathic disease models should be conducted to expand knowledge about luteolin's efficacy, methods enhancing its bioavailability, mechanism of its direct analgesic action, and to ascertain its safety. Clinical trials specifically using luteolin as a complementary analgesic strategy in chronic inflammatory and neuropathic conditions are necessary to establish an effective and safe dose for patients.

In conclusion, luteolin is considered a very promising, safe, and effective natural compound in the management of pain, both in chronic inflammatory conditions and neuropathy. Further studies, especially clinical trials, are necessary to establish its role as an adjuvant agent in therapeutic protocols.
