# Supplementary material for: Luteolin: A promising natural agent in management of pain in chronic conditions
Source: Front Pain Res (Lausanne). 2023 Mar 1;4:1114428. doi: 10.3389/fpain.2023.1114428 (PMC10016360; doi:10.3389/fpain.2023.1114428)
Supplement: Supplementary file 1 [file Table1.docx]

**Luteolin: A Promising Natural Agent in Management of Pain in Chronic Conditions**

**Foteini Ntalouka*, Athina Tsirivakou**

*** Correspondence:** fntalouka@gmail.com

***Supplementary Material***

# Supplementary Table

Table S1 contains a comparative list of all previous studies with the plants that contain luteolin.

| **Family** | **Species** | **Aglycone/Glycoside** | **Rf** |
| --- | --- | --- | --- |
| Anacardiaceae | *Pistacia lentiscus* | luteolin | [107,108] |
| Annonaceae | *Annona tomentosa* | luteolin-7-O-glucoside | [15] |
| Annonaceae | *Cymbopetalum brasiliense* | luteolin-6-hydroxy-7-O-rhamnosylglucoside | [15] |
| Apiaceae | *Cuminum cyminum* | Luteolin 7-galacturonide-4'-glucoside | [109] |
| Apiaceae | *Apium graveolens* | Luteolin 3'-methylether 7-apiosyl-(1->2)-glucoside | [109] |
| Apiaceae | *Angelica keiskei* | cynaroside (luteolin 7-O-D-glucoside) | [110] |
| Apiaceae | *Anthriscus sylvestris* | luteolin-7-O-glucoside | [111] |
| Apiaceae | *Apium graveolens* | luteolin 7-O-apiosylglucoside, luteolin 7-O-glucoside, malonyl derivatives of these glycosides. | [112] |
| Apiaceae | *Bupleurum flavum* | luteolin | [113] |
| Apiaceae | *Chaerophyllum hirsutum* | 6-methylluteolin, luteolin-7-O-beta-D-glucopyranoside | [114] |
| Apiaceae | *Daucus carota* | luteolin, luteolin 3'-O-beta-D-glucopyranoside and luteolin 4'-O-beta-Dglucopyranoside | [115] |
| Apiaceae | *Washingtonia filifera* | luteolin 7-O-glucoside 4''-sulfate (2), luteolin 7-O-glucoside 2''-sulfate | [116] |
| Araceae | *Arum palaestinum* | isoorientin, luteolin | [15] |
| Arecaceae | *Pratia nummularia* | luteolin 7-O-rutinoside, luteolin-7-O-beta-D-rutinoside,luteolin 7-rutinoside | [109] |
| Arecaceae | *Opsiandra maya* | luteolin 7-sulfate-3'-rutinoside | [109] |
| Arecaceae | *Aiphanes aculeata* | luteolin | [117] |
| Arecaceae | *Phoenix dactylifera* | rhamnosyl diglucosyl luteolin, diglucosyl luteolin sulphate, rhamnosyldiglucosyl methyl luteolin, , rhamnosyl glucosyl methyl luteolin, glucosylluteolin sulphate, glucosyl methyl luteolin sulphate | [15] |
| Asclepiadaceae | *Caralluma attenuata* | luteolin-4'-O-neohesperidoside = (luteolin-4'-O-[alpha-(L-rhamnopyranosyl-(1-->2)-beta-D-glucopyranoside)]) | [67] |
| Asclepiadaceae | *Caralluma negevensis* | luteolin 3'-O-beta-D-glucopyranoside-4'-O-alpha-L-rhamnopyranosyl-(1-->2)-beta-D-glucopyra noside, luteolin 3',4'-di-O-beta-D-glucopyranoside | [67] |
| Asclepiadaceae | *Caralluma russeliana* | luteolin 4'-O-beta-D-neohesperidoside. | [15] |
| Asclepiadaceae | *Caralluma tuberculata* | luteolin.4'-beta-D-glucopyranosyl-(2-->1)-alpha-L-rhamnopyranoside | [15] |
| Asclepiadaceae | *Stapelia hirsuta L.* | luteolin-7-O-beta-D-glucopyranoside | [119] |
| Asphodelaceae | *Aloe vera* | luteolin | [120] |
| Aspleniaceae | *Asplenium normale* | luteolin 7-O-dirhamnoside, luteolin 7-O-glucosylrhamnoside | [121] |
| Asteraceae | *Phoenix roebelenii* | luteolin 7-O-rutinoside, luteolin-7-O-beta-D-rutinoside,luteolin 7-rutinoside | [109] |
| Asteraceae | *Launaea nudicaulis* | luteolin 3',7-di-O-beta-glucoside or luteolin 7,3'-diglucoside | [109] |
| Asteraceae | *Launaea nudicaulis* | luteolin 7,4'-diglucoside | [109] |
| Asteraceae | *Vernonia baldwinii* | luteolin 7-galactoside-4'-glucoside | [109] |
| Asteraceae | *Achillea millefolium* | luteolin | [122] |
| Asteraceae | *Achillea nobilis* | luteolin-6-C-apiofuranosyl-(1'''-->2'')-glucoside, orientin, isoorientin, luteolin-luteolin-6-C-apiofuranosyl-(1'''-->2'')-glucoside, orientin, isoorientin, luteolin- 7-O-beta-glucuronide, luteolin-4'-O-beta-glucoside | [15] |
| Asteraceae | *Achillea pannonica* | luteolin-7-O-glucopyranoside, luteolin-7,4'-O-beta-diglucoside | [123] |
| Asteraceae | *Achyrocline satureioides* | luteolin | [15] |
| Asteraceae | *Anthemis chia* | luteolin-7-glucoside | [15] |
| Asteraceae | *Anthemis cretica* | 6- hydroxyluteolin 6- methyl ether, 6- hydroxyluteolin 6,3’ - dimethyl ether,6- hydroxyluteolin 6,7,4’ - trimethyl ether, luteolin-7-glucoside | [15] |
| Asteraceae | *Anthemis monantha* | 6- hydroxyluteolin 6,7,4’ - trimethyl ether, luteolin-7-glucuronide, luteolin-7-glucoside | [15] |
| Asteraceae | *Arnica montana* | luteolin 3'- O-beta-glucoside; | [15] |
| Asteraceae | *Artemisia giraldii* | luteolin | [124] |
| Asteraceae | *Artemisia montana* | luteolin 7-O-rutinoside (scolymoside) | [125] |
| Asteraceae | *Bidens tripartita* | cynaroside, luteolin | [126] |
| Asteraceae | *Carduus crispus* | luteolin-7-glucoside | [127] |
| Asteraceae | *Carduus micropterus* | luteolin | [128] |
| Asteraceae | *Carthamus lanatus* | luteolin 7-O-glucoside | [15] |
| Asteraceae | *Carthamus tinctorius* | luteolin, luteolin 7-O-beta-D-glucopyranoside, luteolin-7-O-(6''-O-acetyl)-beta-D-glucopyranoside | [129] |
| Asteraceae | *Centaurea scoparia* | luteolin | [130] |
| Asteraceae | *Chamomilla recutita* | luteolin, luteolin-7-O-glucoside | [131] |
| Asteraceae | *Chromolaena odorata* | luteolin | [132] |
| Asteraceae | *Chrysanthemum morifolium* | luteolin-7-O-beta-D-glucoside, luteolin | [15] |
| Asteraceae | *Chrysanthemum segetum* | luteolin-7-glucoside | [15] |
| Asteraceae | *Chrysanthemum sinense* | luteolin | [133] |
| Asteraceae | *Cirsium japonicum* | luteolin | [134] |
| Asteraceae | *Cirsium rivulare* | luteolin | [135] |
| Asteraceae | *Conyza bonariensis* | luteolin | [136] |
| Asteraceae | *Cynara scolymus* | luteolin, luteolin-7-O-glucoside, luteolin-7-rutinoside, cynaroside | [15] |
| Asteraceae | *Eclipta alba* | luteolin | [15] |
| Asteraceae | *Erigemn acris* | luteolin | [137] |
| Asteraceae | *Euterpe oleraceae* | orientin | [138] |
| Asteraceae | *Glossogyne tenuifolia* | luteolin, luteolin-7-glucoside | [139] |
| Asteraceae | *Helichrysum compactum* | luteolin, luteolin-7-O-glucoside, luteolin-4',7-di-O-glucoside | [140] |
| Asteraceae | *Helichrysum pamphylicum* | luteolin, luteolin-4’glucoside | [15] |
| Asteraceae | *Inula britannica* | luteolin | [141] |
| Asteraceae | *Ismelia versicolor* | luteolin-7-glucoside, luteolin-7-glucuronide | [15] |
| Asteraceae | *Ixeridium gracile* | luteolin 7-O-glucoside | [142] |
| Asteraceae | *Ixeris denticulata* | luteolin-7-O-glucoside, luteolin-7-O-glucuronide-6'-methyl ester | [143] |
| Asteraceae | *Ixeris sonchifolia* | luteolin, luteolin 7-glucuronide methylester, luteolin 7-glucuronide ethylester,luteolin 7-glucoside, luteolin 7-glucopyranosyl-(1-->6)-glucoside, luteolin 7-glucopyranosyl-(1-->2)-glucoside | [15] |
| Asteraceae | *Lactuca indica* | luteolin, luteolin 7-O-glucuronide | [144] |
| Asteraceae | *Lactuca sativa* | luteolin 7-O-glucuronide | [145] |
| Asteraceae | *Lactuca scariola* | luteolin-7-O-beta-D-glucopyranoside, luteolin | [146] |
| Asteraceae | *Leontodon croceus* | luteolin, luteolin 7-O-beta-D-gentiobioside, luteolin 7-O-beta-D-glucoside, luteolin 7-O-beta-D-glucuronide, luteolin 4'-O-beta-D-glucoside | [15] |
| Asteraceae | *Leontodon duboisii* | luteolin, luteolin 7-O-beta-D-gentiobioside, luteolin 7-O-beta-D-glucoside, luteolin 7-O-beta-D-glucuronide, luteolin 4'-O-beta-D-glucoside | [15] |
| Asteraceae | *Leontodon helveticus* | luteolin, luteolin 7-O-beta-D-gentiobioside, luteolin 7-O-beta-D-glucoside, luteolin 7-O-beta-D-glucuronide, luteolin 4'-O-beta-D-glucoside | [15] |
| Asteraceae | *Leontodon montaniformis* | luteolin, luteolin 7-O-beta-D-gentiobioside, luteolin 7-O-beta-D-glucoside, luteolin 7-O-beta-D-glucuronide | [15] |
| Asteraceae | *Leontodon autumnalis* | luteolin, luteolin 7-O-beta-D-gentiobioside, luteolin 7-O-beta-D-glucoside, luteolin 7-O-beta-D-glucuronide, luteolin 4'-O-beta-D-glucoside | [15] |
| Asteraceae | *Leontodon pyrenaicus* | luteolin, luteolin 7-O-beta-D-gentiobioside, luteolin 7-O-beta-D-glucoside, luteolin 7-O-beta-D-glucuronide, luteolin 4'-O-beta-D-glucoside | [15] |
| Asteraceae | *Leontodon rilaensis* | luteolin, luteolin 7-O-beta-D-gentiobioside, luteolin 7-O-beta-D-glucoside, luteolin 7-O-beta-D-glucuronide, luteolin 4'-O-beta-D-glucoside | [15] |
| Asteraceae | *Leontopodium alpium* | luteolin-7-O-beta-D-glucoside, luteolin-3'-O-beta-D-glucoside, luteolin-4'-O-beta-D-glucoside, 6-hydroxy-luteolin-7-O-beta-D-glucoside, luteolin-7,4'-di-O-beta-D-glucoside | [147] |
| Asteraceae | *Leucanthemum adjustum* | luteolin-7-glucoside, luteolin-7-glucuronide | [15] |
| Asteraceae | *Leucanthemum vulgare* | luteolin-7-glucuronide | [15] |
| Asteraceae | *Lychnophora pohlii* | luteolin | [148] |
| Asteraceae | *Phagnalon rupestre* | luteolin-7-O-beta-glucoside, luteolin-7-O-beta-glucuronide | [149] |
| Asteraceae | *Picnomon acarna* | luteolin-7, 3'-dimethyl ether and luteolin-3'-methyl ether | [150] |
| Asteraceae | *Santolina insularis* | luteolin and luteolin 7-O-beta-D-glucopyranoside | [151] |
| Asteraceae | *Saussurea tridactyla* | luteolin, luteolin-7-O-beta-D-glucoside | [152] |
| Asteraceae | *Scorzonera austriaca* | luteolin 3'-(6-E-p-coumaroyl-beta-d-glucopyranoside) | [15] |
| Asteraceae | *Serratula coronata* | luteolin, luteolin 4'beta-D-glucoside | [153] |
| Asteraceae | *Stevia rebaudiana* | luteolin | [154] |
| Asteraceae | *Tanacetum vulgaris* | luteolin, 6-hydroxyluteolin 6-methyl ether, 6-hydroxyluteolin 6,3'-dimethylether, 6-hydroxyluteolin 6-hydroxyluteolin | [15] |
| Asteraceae | *Tanacetum parthenium* | luteolin 7-glucuronide, luteolin 7-glucoside, luteolin | [15] |
| Asteraceae | *Taraxacum officinale* | luteolin 7-glucoside, luteolin 7-diglucosides, luteolin | [155] |
| Asteraceae | *Tripleurospermum maritimum* | luteolin, luteolin-7-glucoside, luteolin-7-glucuronide | [15] |
| Asteraceae | *Tripleurospermum perforatum* | luteolin, luteolin-7-glucoside | [15] |
| Asteraceae | *Vernoniopsis caudatawith* | luteolin 4'-beta-d-O-glucopyranosyl | [15] |
| Asteraceae | *Wedelia paludosa* | luteolin | [66] |
| Asteraceae | *Youngia japonica* | luteolin-7-O-glucoside | [156] |
| Avicenniaceae | *Avicennia marina* | luteolin 7-O-methylether, luteolin 7-O-methylether 3'-O-beta-D-glucoside, luteolin 7-O-methylether 3'-O-beta-D-galactoside | [66] |
| Balsaminaceae | *Impatiens textori* | luteolin | [158] |
| Begoniaceae | *Begonia malabarica* | luteolin | [15] |
| Berberidaceae | *Epimedium hunanense* | luteolin | [159] |
| Berberidaceae | *Epimedium sagittatum* | luteolin | [160] |
| Bignoniaceae | *Newbouldia laevis* | luteolin | [161] |
| Bignoniaceae | *Tecoma stans Juss.* | luteolin 7-O-beta-D-neohespridoside, luteolin 7-O-beta-D-glucopyranoside | [15] |
| Boraginaceae | *Ehretia ovalifolia* | luteolin | [15] |
| Brassicaceae | *Pratia nummularia* | luteolin 7-O-rutinoside, luteolin-7-O-beta-D-rutinoside,luteolin 7-rutinoside | [109] |
| Brassicaceae | *Brassica napus* | luteolin | [162] |
| Bromeliaceae | *Vriesea sanguinolenta* | 6-hydroxyluteolin-7-O-(1"-alpha-rhamnoside) | [163] |
| Buddlejaceae | *Buddleja globosa* | luteolin-7-O-glucoside | [164] |
| Buddlejaceae | *Buddleja officinalis* | luteolin, luteolin, luteolin-7-O-beta-D-glucopyranoside | [15] |
| Caesalpiniaceae | *Bauhinia tarapotensis* | luteolin 4'-O-beta-D-glucopyranoside | [15] |
| Caesalpiniaceae | *Cassia nigricans* | luteolin | [165] |
| Caesalpiniaceae | *Ceratonia siliqua* | luteolin | [107] |
| Caesalpiniaceae | *Senna petersiana* | luteolin | [166] |
| Caesalpiniaceae | *Senna siamea* | luteolin | [167] |
| Caesalpiniaceae | *Tamarindus indica L.* | luteolin | [168] |
| Campanulaceae | *Capsella bursa-pastoris* | luteolin 7-O-rutinoside, luteolin-7-O-beta-D-rutinoside,luteolin 7-rutinoside | [109] |
| Campanulaceae | *Campanula rotundifolia* | luteolin 7-gentiobioside | [109] |
| Campanulaceae | *Campanula patula* | luteolin 7-(3'''-acetylapiosyl-(1->2)-xyloside) | [109] |
| Campanulaceae | *Lobelia chinensis* | luteolin | [169] |
| Campanulaceae | *Platycodon grandiflorum* | luteolin, luteolin 7-O-glucoside | [15] |
| Cannabaceae | *Cannabis sativa* | orientin, luteolin-7-O-beta-D-glucuronide | [170] |
| Capparaceae | *Capparis himalayensis* | luteolin | [171] |
| Capparaceae | *Capparis spinosa* | luteolin 7-O-glucoside, luteolin | [172] |
| Caprifoliaceae | *Lonicera confusa* | luteolin | [173] |
| Caprifoliaceae | *Lonicera japonica* | luteolin, luteolin 7-O-beta-D-glucopyranoside | [15] |
| Caryophyllaceae | *Dacrydium spp.* | luteolin 7-arabinopyranosyl-(1->6)-glucoside | [174] |
| Caryophyllaceae | *Dianthus chinensis* | isoorientin-2"-O-glucoside | [175] |
| Caryophyllaceae | *Gypsophila repens* | luteolin-7-O-alpha-L-arabinopyranosyl-6-C-beta-glucopyranoside | [15] |
| Caryophyllaceae | *Lychnis flos-cuculi* | luteolin, luteolin 8-C-beta-D: -glucopyranoside | [15] |
| Cecropiaceae | *Cecropia lyratiloba* | isoorientin | [15] |
| Cecropiaceae | *Cecropia obtusifolia* | isoorientin | [176] |
| Chenopodiaceae | *Cornulaca monacantha* | luteolin-7-O-rhamnoside, luteolin-7-O-glucoside | [177] |
| Clusiaceae | *Hypericum brasiliense* | luteolin | [178] |
| Clusiaceae | *Hypericum perforatum* | isoorientin | [179] |
| Colchicaceae | *Colchicum speciosum* | luteolin 7-laminaribioside | [109] |
| Colchicaceae | *Colchicum cilicicum* | luteolin | [180] |
| Combretaceae | *Terminalia arjuna* | luteolin | [181] |
| Combretaceae | *Terminalia chebula retz.* | luteolin | [182] |
| Combretaceae | *Terminalia myriocarpa* | orientin, isoorientin | [183] |
| Commelinaceae | *Commelina communis* | orientin, isoorientin | [15] |
| Conocephalaceae | *Conocephalum Coicum* | luteolin 7-glucuronide-4'-rhamnoside | [109] |
| Cucurbitaceae | *Cucumis sativus* | luteolin-8-C-beta-D-glucopyranoside (orientin), luteolin-6-C-beta-Dglucopyranoside (isoorientin) | [15] |
| Cucurbitaceae | *Luffa cylindrica* | luteolin-7-O-beta-D-glucuronide methyl ester | [184] |
| Cucurbitaceae | *Sechium edule* | luteolin glycosides | [185] |
| Cynomoriaceae | *Cynomorium songaricum* | luteolin | [186] |
| Cyperaceae | *Cyperus alopecuroides* | luteolin 5,3'-dimethylether, orientin | [15] |
| Cyperaceae | *Cyperus conglomeratus* | luteolin, luteolin 7-methyl ether | [15] |
| Dicranaceae | *Dicranum scoparium* | luteolin-7-O-neohesperidoside | [187] |
| Equisetaceae | *Equisetum arvense* | luteolin | [188] |
| Euphorbiaceae | *Chrozophora brocchiana* | luteolin 7-O-glucoside | [189] |
| Euphorbiaceae | *Glochidion zeylanicum* | isoorientin | [190] |
| Euphorbiaceae | *Jatropha cilliata* | isoorientin and orientin | [15] |
| Euphorbiaceae | *Phyllanthus emblica* | luteolin-4'-O-neohesperiodoside | [191] |
| Fabaceae | *Vicia balansae* | luteolin 7,4'-diglucoside | [109] |
| Fabaceae | *Dalbergia monetarya* | luteolin 3'-methylether 7-apiosyl-(1->2)-glucoside | [109] |
| Fabaceae | *Cajanus cajan* | luteolin | [192] |
| Fabaceae | *Crotalaria sessiliflora* | isoorientin, orientin | [193] |
| Fabaceae | *Dioclea lasiophylla* | luteolin 3'beta-D-glucopyranoside | [194] |
| Fabaceae | *Spartium junceum L.* | luteolin 4'-beta-glucoside | [195] |
| Flacourtiaceae | *Homalium brachybotrys* | luteolin-7-O-beta-glucopyranoside | [196] |
| Flacourtiaceae | *Hydnocarpus wightiana* | luteolin | [197] |
| Gentianaceae | *Gentiana algida* | orientin | [15] |
| Gentianaceae | *Gentiana arisanensis* | luteolin-7-O-beta-D-glucoside, isoorientin-6"-O-glucoside | [15] |
| Gentianaceae | *Gentiana olivieri* | isoorientin | [198] |
| Gentianaceae | *Gentiana piasezkii* | isoorientin, luteolin | [199] |
| Gentianaceae | *Gentianella nitida* | isoorientin | [200] |
| Gentianaceae | *Lisianthius nigrescens* | luteolin 8-C-glucoside | [201] |
| Gentianaceae | *Swertia punctata* | isoorientin | [15] |
| Geraniaceae | *Biebersteinia orphanidis* | luteolin | [202] |
| Geraniaceae | *Pelargonium reniforme* | 2''-O-galloylisoorientin | [15] |
| Ginkgoaceae | *Ginkgo biloba* | luteolin | [15] |
| Globulariaceae | *Globularia alypum* | 6-hydroxyluteolin 7-O-laminaribioside, 6-hydroxyluteolin 7-O-beta-Dglucopyranoside, luteolin 7-O-sophoroside | [15] |
| Hedwigiaceae | *Hedwigia ciliata* | luteolin 7-O-Neohesperidoside-4'-O-sophoroside | [15] |
| Hydrocharitaceae | *Elodea canadensis* | luteolin 7-glucuronosyl-(1->2)-glucuronide | [109] |
| Hydrocharitaceae | *Elodea nuttallii* | luteolin-7-O-diglucuronide | [203] |
| Jubulaceae | *Frullania dilatata* | luteolin 7,4'-diglucoside | [109] |
| Labiatae | *Sideritis maura* | luteolin 7-allosyl-(1->2)-glucoside | [109] |
| Labiatae | *Salvia triloba* | luteolin 7-glucuronide-3'-glucoside | [204] |
| Labiatae | *Glechoma hederacea* | luteolin 7-O-beta-D-glucopyranoside | [205] |
| Labiatae | *Lycopus lucidus* | luteolin, luteolin-7-O-beta-D-glucuronide methyl ester | [206] |
| Labiatae | *Salvia palaestina* | luteolin, luteolin glycosides | [207] |
| Labiatae | *Salvia sclarea* | luteolin | [208] |
| Lamiaceae | *Thymus membraneceus* | luteolin 7- sambubioside | [109] |
| Lamiaceae | *Saussurea medusa* | luteolin 7-O-rutinoside, luteolin-7-O-beta-D-rutinoside, luteolin 7-rutinoside | [109] |
| Lamiaceae | *Ajuga genevensis* | luteolin | [209] |
| Lamiaceae | *Ajuga reptans* | luteolin | [210] |
| Lamiaceae | *Ballota nigra* | luteolin-7-lactate, luteolin-7-glucosyl-lactate | [15] |
| Lamiaceae | *Coleus parvifolius* | luteolin, luteolin 5-O-beta-d-glucopyranoside, luteolin 7-methyl ether, luteolin 5-O-beta-d-glucuronide, 5-O-beta-d-glucopyranosyl-luteolin 7-methyl ether | [211] |
| Lamiaceae | *Dracocephalum subcapitatum* | luteolin | [212] |
| Lamiaceae | *Elsholtzia blanda* | luteolin | [213] |
| Lamiaceae | *Elsholtzia bodinieri* | luteolin 7-O-[6''-(3'''-hydroxy-4'''-methoxy cinnamoyl)]-beta-Dglucopyranoside | [15] |
| Lamiaceae | *Elsholtzia rugulosa* | luteolin, luteolin 3'-glucuronyl acid methyl ester | [214] |
| Lamiaceae | *Lamiophlomis rotata* | luteolin-7-O-glucoside, luteolin | [215] |
| Lamiaceae | *Lavandula stoechas* | luteolin 7-O-glucoside | [216] |
| Lamiaceae | *Lepechinia graveolens* | luteolin-7-O-glucuronide | [217] |
| Lamiaceae | *Leucas cephalotes* | luteolin 4'-O-beta-D-glucuronopyranoside | [15] |
| Lamiaceae | *Lycopus europaeus* | luteolin-7-glucoside, luteolin-7-glucuronide | [218] |
| Lamiaceae | *Lycopus virginicus* | luteolin | [219] |
| Lamiaceae | *Melissa officinalis* | luteolin, luteolin 7-O-beta-D-glucopyranoside, luteolin 7-O-beta-Dglucuronopyranoside, luteolin 3'-O-beta-D-glucuronopyranoside, luteolin 7-O-beta-D-glucopyranoside-3'-Obeta-D-glucuronopyranoside | [15] |
| Lamiaceae | *Mentha x piperita* | luteolin-7-O-rutinoside | [15] |
| Lamiaceae | *Nepeta cataria* | luteolin 7-O-glucuronide, luteolin 7-O-glucurono-(1-->6)-glucoside, luteolin | [220] |
| Lamiaceae | *Nepeta sibthorpii* | luteolin-7-O-glucoside | [15] |
| Lamiaceae | *Ocimum gratissimum* | luteolin | [221] |
| Lamiaceae | *Ocimum sanctum* | luteolin-7-O-beta-D-glucuronic acid 6''-methyl ester, luteolin-7-O-beta-Dglucopyranoside, luteolin-5-O-beta-D-glucopyranoside | [15] |
| Lamiaceae | *Origanum vulgare* | luteolin | [222] |
| Lamiaceae | *Perilla frutescens* | luteolin, luteolin 7-O-glucuronide-6"-methyl ester | [15] |
| Lamiaceae | *Perilla ocimoides* | luteolin 7-0-(glucuronosyl(beta-l-->-2-glucuronide) | [223] |
| Lamiaceae | *Phlomis aurea* | luteolin-7-O-beta-glucopyranoside | [224] |
| Lamiaceae | *Phlomis brunneogaleata* | luteolin 7- O-beta- D-glucopyranoside | [225] |
| Lamiaceae | *Phlomis lunariifolia* | luteolin 7-O-[4-O-acetyl-alpha-rhamnopyranosyl-(1-->2)]-betaglucuronopyranoside | [226] |
| Lamiaceae | *Rosmarinus officinalis* | luteolin 3'-O-beta-D-glucuronide, luteolin 3'-O-(4"-O-acetyl)-beta-Dglucuronide,luteolin 3'-O-(3"-O-acetyl)-beta-D-glucuronide | [15] |
| Lamiaceae | *Salvia officinalis* | luteolin 7-O-beta-D-glucoside, luteolin 7--O-beta-D-glucuronide, luteolin 3'-O-beta-D-glucuronide, 6-hydroxyluteolin 7-O-beta-D-glucoside, 6-hydroxyluteolin 7-O-glucuronide | [15] |
| Lamiaceae | *Satureja obovata* | luteolin | [227] |
| Lamiaceae | *Satureja parvifolia* | luteolin | [15] |
| Lamiaceae | *Schizonepeta tenuifolia* | luteolin | [228] |
| Lamiaceae | *Scutellaria barbata* | luteolin | [229] |
| Lamiaceae | *Scutellaria species* | luteolin | [15] |
| Lamiaceae | *Teucrium species* | luteolin | [230] |
| Lamiaceae | *Thymus broussonettii* | luteolin, luteolin-7-O-glucoside, luteolin-3'-O-glucuronide | [15] |
| Lamiaceae | *Thymus piperella* | luteolin-7- O-beta- D-glucoside | [15] |
| Lamiaceae | *Thymus vulgaris* | luteolin 7-glucuronide | [231] |
| Lamiaceae | *Thymus willdenowii* | luteolin-3'-O-glucuronide | [232] |
| Lamiaceae | *Vitex agnus-castus* | luteolin 6-C-(4"-methyl-6"-O-trans-caffeoylglucoside), luteolin 6-C-(6"-Otrans-caffeoylglucoside), luteolin 6-C-(2"-O-trans-caffeoylglucoside), luteolin 7-O-(6"-p-benzoylglucoside), luteolin, orientin | [15] |
| Lamiaceae | *Vitex polygama* | orientin, isoorientin | [15] |
| Lamiaceae | *Vitex rotundifolia* | luteolin | [233] |
| Lamiaceae | *Zataria multiflora* | luteolin | [234] |
| Ledocarpaceae | *Balbisia calycina* | luteolin | [235] |
| Leguminosae | *Aspalathus linearis* | luteolin, orientin | [15] |
| Leguminosae | *Cyclopia subternata* | luteolin | [236] |
| Leguminosae | *Genista corsica* | luteolin, luteolin 4'-O-beta-glucoside, luteolin 7-O-beta-glucoside | [237] |
| Leguminosae | *Genista morisii* | luteolin, luteolin 7-O-beta-D-glucopyranoside, luteolin 4'-O-beta-Dglucopyranoside | [15] |
| Leguminosae | *Genista tenera* | luteolin-7-O-glucoside, luteolin-7,3'-di-O-glucoside | [238] |
| Leguminosae | *Glycine soja* | luteolin, luteolin-7-O-beta-D-glucoside | [239] |
| Leguminosae | *Kummerowia striata* | luteolin 4'-O-glucopyranoside | [240] |
| Leguminosae | *Medicago sativa* | luteolin, luteolin 7-O-[2-O-feruloyl-beta-D-glucuronopyranosyl(1-->2)-Obeta-D-glucuronopyranosyl]-4 '-O-beta-D-glucuronopyranoside, luteolin 7-O-beta-D-glucuronopyranoside | [15] |
| Leguminosae | *Medicago truncatula* | luteolin 7-O-[beta-D-glucuronopyranosyl-(1-->2)]-O-beta-Dglucuronopyranoside,luteolin 7-O-beta-D-glucuronopyranoside | [241] |
| Leguminosae | *Retama raetam* | luteolin 4'-O-neohesperidoside | [242] |
| Leguminosae | *Retama sphaerocarpa* | orientin | [243] |
| Lythraceae | *Cuphea pinetorum* | luteolin-7-O-beta-D-glucopyranoside | [15] |
| Lythraceae | *Lawsonia inermis* | luteolin | [244] |
| Lythraceae | *Lythrum salicaria* | isoorientin, orientin | [245] |
| Lythraceae | *Punica granatum* | luteolin | [246] |
| Malvaceae | *Abutilon indicum* | luteolin, luteolin 7-O-beta-glucopyranoside | [247] |
| Malvaceae | *Kitaibelia vitifolia* | luteolin | [248] |
| Marchantiaceae | *Marchantia polymorpha* | luteolin 7,3'- diglucuronide | [109] |
| Marchantiaceae | *Marchantia berteroana* | luteolin 7,3'-digalacturonide, luteolin 3',4'- digalacturonide | [249] |
| Marchantiaceae | *Marchantia polymorpha* | luteolin 7,4'-diglucuronide | [109] |
| Melanthiaceae | *Japonolirion osense* | isoorientin, orientin | [250] |
| Melastomataceae | *Leandra lacunosa* | luteolin | [15] |
| Mimosaceae | *Albizzia julibrissin* | luteolin | [15] |
| Monocleaceae | *Monoclea forsteri* | 6-methoxyluteolin 7-O-[2-O-alpha-rhamnosyl-3-O-alpha-arabinosyl-betaglucuronide]-4'-O-[2-O-alpha-r hamnosyl-3-O-beta-xylosyl-betaglucuronide],6-methoxyluteolin 7-O-[2-O-alpha-rhamnosyl-betaglucuronide]-4'-O-[2-O-alpha-rhamnosyl-3-O-beta-xylosyl-betaglucuronide]. | [251] |
| Moraceae | *Broussonetia papyrifera* | luteolin | [15] |
| Moraceae | *Ficus carica* | luteolin | [107] |
| Ochnaceae | *Lophira alata* | luteolin | [252] |
| Oleaceae | *Ligustrum vulgare* | luteolin, luteolin 7-O-glucoside | [253] |
| Oleaceae | *Olea europaea* | luteolin, luteolin-4'-O-glucoside, luteolin-7-O-glucoside | [15] |
| Oleaceae | *Phillyrea latifolia* | luteolin, luteolin 7-O-glucoside, luteolin 4’-O-glucoside | [253] |
| Ophioglossaceae | *Lunularia cruciata* | luteolin 3',4'- diglucuronide | [109] |
| Ophioglossaceae | *Ophioglossum petiolatum* | luteolin | [254] |
| Orchidaceae | *Listera ovata* | luteolin 3',4'- diglucoside | [255] |
| Oxalidaceae | *Oxalis corniculata* | isoorientin | [256] |
| Oxalidaceae | *Oxalis triangularis* | luteolin 6-C-(2''-O-beta-xylopyranosyl-beta-glucopyranoside) | [15] |
| Paeoniaceae | *Paeonia suffruticosa* | luteolin-7-O-glucoside | [257] |
| Papaveraceae | *Papaver rhoeas* | luteolin | [258] |
| Passifloraceae | *Passiflora alata* | 2’’-rhamnosyl-orientin, isoorientin, orientin | [15] |
| Passifloraceae | *Passiflora caerulea* | isoorientin, orientin | [15] |
| Passifloraceae | *Passiflora edulis* | luteolin -7-O-[2-rhamnosylglucoside], isoorientin, orientin, luteolin glycosides | [259] |
| Passifloraceae | *Passiflora incarnata* | isoorientin-2''-O-glucopyranoside, isoorientin, orientin | [15] |
| Piperaceae | *Piper solmsianum* | orientin | [260] |
| Plantaginaceae | *Veronicastrum sibir-icum* | Lonicerin / luteolin 7- neohesperidoside | [109] |
| Plantaginaceae | *Penstemon gentianoides* | luteolin | [261] |
| Plantaginaceae | *Plantago lagopus* | luteolin-7-O-beta-glucoside | [15] |
| Plantaginaceae | *Plantago lanceolata* | luteolin | [262] |
| Plantaginaceae | *Plantago maritima* | luteolin | [263] |
| Poaceae | *Cymbopogon citraee* | Lonicerin / luteolin 7- neohesperidoside | [109] |
| Poaceae | *Cymbopogon citratus* | isoorientin, isoorientin 2' '-O-rhamnoside, orientin | [264] |
| Poaceae | *Deschampsia antarctica* | luteolin, orientin, orientin 2''-O-arabinopyranoside, isoswertiajaponin (7-Omethylorientin),isoswertiajaponin 2''-O-beta-arabinopyranoside | [15] |
| Poaceae | *Deschampsia borealis* | orientin | [15] |
| Poaceae | *Digitaria exilis* | luteolin | [265] |
| Poaceae | *Echinochloa utilis* | luteolin | [266] |
| Poaceae | *Hordeum vulgare* | isoorientin, , isoorientin-7-O-glucoside | [15] |
| Poaceae | *Phyllostachys nigra* | luteolin-7-O-glucoside, luteolin 6-C-(6''-O-trans-caffeoylglucoside) | [15] |
| Poaceae | *Pogonatherum crinitum* | luteolin 6-C-beta-boivinopyranoside, luteolin, luteolin 6-C-betafucopyranoside, luteolin 6-C-beta-glucopyranoside | [267] |
| Poaceae | *Saccharum officinarum* | orientin, luteolin-8-C-(rhamnosylglucoside), 4',5'-dimethyl-luteolin-8-Cglycoside | [268] |
| Poaceae | *Sasa borealis* | isoorientin, isoorientin 2"-O-alpha-L-rhamnoside | [15] |
| Poaceae | *Secale cereale* | luteolin 7-O-diglucuronyl-4'-O-glucuronide, luteolin 7-O-diglucuronide | [269] |
| Poaceae | *Setaria viridis* | orientin 2''-O-xyloside | [270] |
| Poaceae | *Triticum durum* | luteolin glycosides | [271] |
| Podocarpaceae | *Dacrydium spp.* | luteolin 7- arabinofuranosyl-(1- >6)-glucoside | [109] |
| Podocarpaceae | *Podocarpus Nivalis* | luteolin 7-glucoside-3'- xyloside | [109] |
| Polygonaceae | *Fagopyrum esculentum* | isoorientin, orientin | [272] |
| Polygonaceae | *Fallopia species* | luteolin glycosides | [273] |
| Polygonaceae | *Rumex induratus* | 6-C-hexosyl-luteolin | [15] |
| Polygonaceae | *Rumex luminiastrum* | orientin | [15] |
| Potamogetonaceae | *Potamogeton ssp* | isoorientin, luteolin 7- O-glucoside, luteolin 7-O-glucuronide, luteolin 3’-Oglucoside, luteolin | [274] |
| Pteridaceae | *Pteris cretica* | luteolin 8-C-rhamnoside-7-O-rhamnoside, luteolin 7-O-robinobioside, luteolin 7-O-rutinoside, luteolin 7-O-glucoside | [15] |
| Pteridaceae | *Pteris multifida* | luteolin 7-Omicron-beta-D: -glucopyranoside, luteolin | [15] |
| Ranunculaceae | *Aquilegia ecalcarata* | luteolin | [275] |
| Ranunculaceae | *Aquilegia vulgaris* | luteolin derivatives | [15] |
| Ranunculaceae | *Ficaria verna* | luteolin 8-C-beta-D-glucopyranoside | [276] |
| Ranunculaceae | *Trollius chinensis* | orientin | [15] |
| Ranunculaceae | *Trollius ledibouri* | orientin | [15] |
| Resedaceae | *Reseda luteola* | luteolin 3',7-di-O-beta-glucoside or luteolin 7,3'- diglucoside | [109] |
| Ricciaceae | *Riccia fluitans* | luteolin 7-glucuronide- 3'-glucoside | [109] |
| Rosaceae | *Chaenomeles sinensis* | luteolin-7-O-beta-D-glucuronide, luteolin-3'-methoxy-4'-O-beta-Dglucopyranoside, luteolin-7-O-beta-D-glucuronide methyl ester | [15] |
| Rosaceae | *Crataegus oxyacantha* | luteolin, luteolin-3', 7-diglucoside | [15] |
| Rosaceae | *Crataegus pentagyna* | isoorientin, orientin, isoorientin-2''-O-rhamnoside, orientin-2''-O-rhamnoside | [15] |
| Rosaceae | *Crataegus x macrocarpa* | luteolin-7-O-beta-D-glucuronide | [277] |
| Rosaceae | *Potentilla multifida* | luteolin-7-O-beta-D-glucuronide | [278] |
| Rubiaceae | *Morinda citrifolia* | luteolin | [279] |
| Rubiaceae | *Morinda morindoides* | luteolin, luteolin-7-O-glucoside | [280] |
| Rutaceae | *Citrus bergamia* | orientin 4'-methyl ether | [281] |
| Salicaceae | *Salix gilgiana* | luteolin-7-O-beta-D-glucuronopyranoside, luteolin | [282] |
| Salicaceae | *Salix matsudana* | luteolin-7-O-d-glucoside, luteolin 7-O-beta-D-glucopyranuronide | [15] |
| Sapindaceae | *Allophyllus edulis* | isoorientin 2"-O-rhamnoside, orientin 2"-O-rhamnoside | [283] |
| Scrophulariaceae | *Bacopa monnieri* | luteolin, luteolin-7-O-beta-glucopyranoside | [15] |
| Scrophulariaceae | *Brandisia hancei* | luteolin | [284] |
| Scrophulariaceae | *Hebe parviflora* | luteolin-7-O-beta-glucoside, luteolin-3’-O-beta-glucoside, luteolin-7, 3’-di-O-beta-glucoside, luteolin-4’-O-beta-glucoside, luteolin-7-O-betaglucuronide,8-hydroxyluteolin-8-beta-glucoside, 8-hydroxyluteolin-7-betaglucoside,6-hydroxyluteolin-7-O-beta-[2-O-beta-xyloglucoside], 6-hydroxyluteolin-7-O-beta-[6-O-beta-xyloglucoside], luteolin-7-O-beta-[6-Oalpha-rhamnosylglucoside], luteolin-7-O-beta-[6-O-beta-xyloglucoside], 4’-O-methylluteolin-7-O-beta-[6-O-beta-xyloglucoside] | [15] |
| Scrophulariaceae | *Hebe stenophylla* | luteolin-7-O-beta-glucoside, luteolin-4’-O-beta-glucoside, 8-hydroxyluteolin-8-beta-glucoside, 8-hydroxyluteolin-7-beta-glucoside, 6-hydroxyluteolin-7-beta-glucoside, 6-hydroxyluteolin-7-O-beta-[2-O-betaxyloxyloside],6-hydroxyluteolin-7-O-beta-[2-O-beta-xyloglucoside], 6-hydroxyluteolin-7-O-beta-[2-O-beta-glucoglucoside], 6-hydroxyluteolin-7-O-beta-[6-O-beta-xyloglucoside] | [15] |
| Scrophulariaceae | *Hebe strictissima* | luteolin-7-O-beta-glucoside, luteolin-4’-O-beta-glucoside, , luteolin-7-Obeta-glucuronide, 8-hydroxyluteolin-7-beta-glucoside, 6-hydroxyluteolin-7-O-beta-[2-O-beta-xyloglucoside], 6-hydroxyluteolin-7-O-beta-[6-O-betaxyloglucoside],luteolin-7-O-beta-[6-O-alpha-rhamnosylglucoside], luteolin-7-O-beta-[6-O-beta-xyloglucoside] | [15] |
| Scrophulariaceae | *Hebe traversti* | luteolin-7-O-beta-glucoside, luteolin-7, 3’-di-O-beta-glucoside, luteolin-4’-O-beta-glucoside, 8-hydroxyluteolin-8-beta-glucoside, 8-hydroxyluteolin-7-beta-glucoside, 6-hydroxyluteolin-7-O-beta-[2-O-beta-xyloxyloside], 6-hydroxyluteolin-7-O-beta-[2-O-beta-xyloglucoside], 6-hydroxyluteolin-7-Obeta-[2-O-beta-glucoglucoside], 4’-O-methylluteolin-7-O-beta-[6-O-betaxyloglucoside] | [15] |
| Scrophulariaceae | *Isoplexis chalcantha* | luteolin, luteolin 7- O-beta- D-glycoside | [285] |
| Scrophulariaceae | *Striga lutea* | luteolin | [286] |
| Scrophulariaceae | *Striga orobanchioides* | luteolin | [287] |
| Scrophulariaceae | *Torenia fournieri* | luteolin-7-O-beta-glucoside | [288] |
| Scrophulariaceae | *Verbascum salviifolium* | luteolin 7-O-glucoside, luteolin 3'-O-glucoside | [15] |
| Scrophulariaceae | *Veronica chamaedrys* | luteolin, apigenin, luteolin-3'-methyl ether | [15] |
| Scrophulariaceae | *Veronica thymoides* | luteolin 7-O-beta-glucopyranoside | [289] |
| Simaroubaceae | *Ailanthus excelsa* | luteolin | [290] |
| Solanaceae | *Capsicum annuum* | luteolin 7-O-beta-D-apiofuranosyl-(1-->2)-beta-D-glucopyranoside, luteolin 7-O-[2-(beta-d-apiofuranosyl)-4-(beta-d-glucopyranosyl)-6-malonyl]-betad-glucopyranoside, malonyl)-glucopyranoside, luteolin 6-C-beta-D-glucopyranoside-8-C-alpha-luteolin-7-O-(2-apiofuranosyl-4-glucopyranosyl-6-malonyl)-glucopyranoside, luteolin 6-C-beta-D-glucopyranoside-8-C-alpha-L-arabinopyranoside | [15] |
| Sterculiaceae | *Theobroma cacao* | luteolin, luteolin 7-O-glucoside, orientin, isoorientin | [291] |
| Thymelaeaceae | *Daphne genkwa* | luteolin, luteolin 7-methyl ether | [292] |
| Thymelaeaceae | *Daphne gnidium* | luteolin-3',7-di-O-glucoside | [293] |
| Thymelaeaceae | *Gnidia involucrata* | isoorientin | [294] |
| Turneraceae | *Turnera diffusa* | luteolin 8-C-E-propenoic acid (1), luteolin 8-C-beta-[6-deoxy-2-O-(alpha-lrhamnopyranosyl)-xylo-hexopyranos-3-uloside] | [295] |
| Umbelliferae | *Dystaenia takeshimana* | luteolin | [296] |
| Urticaceae | *Urtica laetevirens* | luteolin 7-O-neohesperidoside, luteolin 7-O-beta-D: -glucopyranoside, 5-methoxyluteolin 7-O-beta-D: -glucopyranoside | [15] |
| Valerianaceae | *Patrinia villosa* | isoorientin | [15] |
| Verbenaceae | *Lippia alba* | luteolin-7-diglucuronide | [15] |
| Verbenaceae | *Verbena officinalis* | luteolin 7-diglucuronide | [15] |
| Verbenaceae | *Aloysia triphylla* | luteolin 7-diglucuronide | [15] |
| Violaceae | *Viola tricolor* | luteolin-6-C-hexoside, luteolin-6-C-deoxyhexoside-8-C-hexoside, luteolin-6-C-hexoside-8-C-deoxyhexoside | [15] |
| Violaceae | *Viola yedoensis* | luteolin 6-C-beta-D-glucopyranoside (isoorientin), luteolin 6-C-alpha-Larabinopyranosyl-8-C-beta-D-glucopyranoside (isocarlinoside) | [15] |
| Vitaceae | *Cayratia japonica* | luteolin, luteolin-7-O-beta-D-glucopyranoside | [15] |
| Zosteraceae | *Zostera marina* | luteolin | [15] |
